# Time-continuous and time-discrete SIR models revisited: theory and applications

**DOI:** 10.1186/s13662-020-02995-1

**Published:** 2020-10-07

**Authors:** Benjamin Wacker, Jan Schlüter

**Affiliations:** 1 Next Generation Mobility Group, Department of Dynamics of Complex Fluids, Max-Planck-Institute for Dynamics and Self-Organization, Am Fassberg 17, D-37077 Göttingen, Germany; 2 Institute for Dynamics of Complex Fluids, Faculty of Physics, Georg-August-University of Göttingen, Friedrich-Hund-Platz 1, D-37077 Göttingen, Germany

**Keywords:** COVID-19, Difference equations, Existence and uniqueness, Mathematical epidemiology, Numerical analysis, Nonlinear ordinary differential equations, SIR model, Well-posedness

## Abstract

Since Kermack and McKendrick have introduced their famous epidemiological SIR model in 1927, mathematical epidemiology has grown as an interdisciplinary research discipline including knowledge from biology, computer science, or mathematics. Due to current threatening epidemics such as COVID-19, this interest is continuously rising. As our main goal, we establish an implicit time-discrete SIR (susceptible people–infectious people–recovered people) model. For this purpose, we first introduce its continuous variant with time-varying transmission and recovery rates and, as our first contribution, discuss thoroughly its properties. With respect to these results, we develop different possible time-discrete SIR models, we derive our implicit time-discrete SIR model in contrast to many other works which mainly investigate explicit time-discrete schemes and, as our main contribution, show unique solvability and further desirable properties compared to its continuous version. We thoroughly show that many of the desired properties of the time-continuous case are still valid in the time-discrete implicit case. Especially, we prove an upper error bound for our time-discrete implicit numerical scheme. Finally, we apply our proposed time-discrete SIR model to currently available data regarding the spread of COVID-19 in Germany and Iran.

## Introduction

### Motivation

Since its outbreak in Wuhan (China) in December 2019, the quick spread of COVID-19 has threatened people worldwide. Politicians around the globe have to balance different interests and need to make tremendous decisions which impact our daily life. For these reasons, governments around the world heavily rely on scientific advice in the present situation. Thus, John Hopkins University has been collecting epidemiological data regarding COVID-19 from many countries during the last months [1, 2]. Additionally, many biological and medical studies regarding different aspects of this new coronavirus have been rapidly appearing in scientific journals [3–9]. However, to estimate the impact of COVID-19, governments need forecasts from as many models as possible.

Kermack and McKendrick introduced the now well-known SIR model in one of mathematical epidemiology’s most groundbreaking works in 1927 [10]. They assumed a fixed population size and divided this population into three different homogeneous groups of people, namely susceptible people, infectious people, and recovered people, excluding births, deaths, and deaths by disease from their model. Due to its success and simplicity, their work was reprinted in 1991 [11–13]. In upcoming decades, epidemiologists and mathematicians have developed many variants and extensions of this basic model by, for example, adding age or spatial structures [14–20].

After the outbreak of COVID-19, many scientists have recently published articles with emphasis on epidemic forecasts which strongly relate to mathematical models. Many approaches, mainly focusing on stochastic arguments, with respect to predicting forecasts of the total number of infected people have appeared during the last weeks [21–26] or in the past [27, 28]. Recently, neural networks have been applied to forecasting [29] and, additionally, different authors such as Atangana, Baleanu, or Khan have used fractional differential equations in extended SIR-type models to investigate the spread of COVID-19 or mathematical biology in general [30–34].

### Contributions

There are works with respect to SIR models [35–37] and their time-discrete versions [38]. However, one finds mainly explicit schemes with respect to time-discrete SIR models in the aforementioned works and references therein. For this reason, we summarize and extend some results on properties of the time-continuous classical SIR model, and we propose an implicit time-discrete version of this classical SIR model and prove that this time-discrete variant maintains many of time-continuous version’s properties. Hence, the aim of our study is to propose a nonautonomous SIR model, analyze the properties of its time-continuous formulation, and develop an implicit numerical solution algorithm where the main properties of the time-continuous variant are conserved. More precisely, our main contributions can be summarized as follows:

1. At first, we propose a modified time-continuous SIR model with time-varying transmission and recovery rates;
2. Secondly, we conclude well-posedness of our time-continuous problem formulation. This includes global existence in time, global uniqueness in time, based on an inductive application of Banach’s fixed point theorem, and continuous dependence on initial conditions and time-varying rates;
3. In case of the time-discrete implicit model, we provide unique solvability, monotonicity properties, and an upper error bound between the solution of the implicit time-discrete problem formulation and the solution of the time-continuous problem formulation;
4. Finally, we compare our model predictions with real-world data regarding the spread of COVID-19 from different countries. Data have been collected by John Hopkins University.

Conclusively, we summarize our results and give a brief outlook on possible extensions.

## Time-continuous SIR model

In this section, we portray the time-continuous SIR model and its properties.

### Mathematical background material

Here, we recall Lipschitz continuity of a function on Euclidean spaces.

#### Definition 1

([39, Sect. 3.2])

Let $d_{1}, d_{2} \in \mathbb{N}$. If $S \subset \mathbb{R}^{d_{1}}$, a defined function $\mathbf{F} \colon S \longrightarrow \mathbb{R}^{d_{2}}$ is called *Lipschitz continuous on* *S* if there exists a nonnegative constant $L \geq 0$ such that

1 $$ \bigl\lVert \mathbf{F} ( \mathbf{x} ) - \mathbf{F} ( \mathbf{y} ) \bigr\rVert _{\mathbb{R}^{d_{2}}} \leq L \cdot \lVert \mathbf{x} - \mathbf{y} \rVert _{\mathbb{R}^{d_{1}}} $$

holds for all $\mathbf{x}, \mathbf{y} \in S$. Here, $\lVert \cdot \rVert $ denotes a suitable norm on the corresponding Euclidean space.

Let $U \subset \mathbb{R}^{d_{1}}$ be open, let $\mathbf{F} \colon U \longrightarrow \mathbb{R}^{d_{2}}$. We shall call **F** *locally Lipschitz continuous* if for every point $\mathbf{x_{0}} \in U$ there exists a neighborhood *V* of $\mathbf{x_{0}}$ such that the restriction of **F** to *V* is Lipschitz continuous on *V*.

In a more general framework, we consider a nonlinear initial value problem

2 $$ \textstyle\begin{cases} \mathbf{z}^{\prime } ( t ) = \mathbf{G} ( t, \mathbf{z} ( t ) ), \\ \mathbf{z} ( 0 ) = \mathbf{z_{0}}, \end{cases} $$

where we define our solution vector $\mathbf{z} ( t ) = ( x_{1} ( t ), \ldots , x_{n} ( t ) )^{T}$, our vectorial function $\mathbf{G} ( t, \mathbf{z} ( t ) ) = ( g_{1} ( t, \mathbf{z} ( t ) ), \ldots , g_{n} ( t, \mathbf{z} ( t ) ) )^{T}$, and our initial condition $\mathbf{z_{0}} \in \mathbb{R}^{n}$. To conclude global existence in time, we can apply the following theorem that is a direct consequence of Grönwall’s lemma.

#### Theorem 1

([39, Theorem 4.2.1])

*If* $\mathbf{G} \colon \mathbb{R}^{n} \longrightarrow \mathbb{R}^{n}$ *is locally Lipschitz continuous and if there exist nonnegative real constants* *B* *and* *K* *such that*

3 $$ \bigl\lVert \mathbf{G} \bigl( t, \mathbf{z} ( t ) \bigr) \bigr\rVert _{\mathbb{R}^{n}} \leq K \cdot \bigl\lVert \mathbf{z} ( t ) \bigr\rVert _{\mathbb{R}^{n}} + B $$

*holds for all* $\mathbf{z} ( t ) \in \mathbb{R}^{n}$, *then the solution of the initial value problem* (2) *exists for all time* $t \in \mathbb{R}$ *and*, *moreover*, *it holds*

4 $$ \bigl\lVert \mathbf{z} ( t ) \bigr\rVert _{\mathbb{R}^{n}} \leq \lVert \mathbf{z_{0}} \rVert _{\mathbb{R}^{n}} \cdot \exp \bigl( K \cdot \vert t \vert \bigr) + \frac{B}{K} \cdot \bigl( \exp \bigl( K \cdot \vert t \vert \bigr) - 1 \bigr) $$

*for all* $t \in \mathbb{R}$.

To prove global uniqueness in time, we need Banach’s fixed point theorem since fixed point theorems have been successfully applied as a versatile tool in different areas of mathematics [40, 41].

#### Theorem 2

([42, Theorem V.18])

*Let* $( X, \varrho )$ *be a complete metric space with the metric mapping* $\varrho \colon X \times X \longrightarrow [ 0, \infty )$. *Let* $T \colon X \longrightarrow X$ *be a* strict contraction, *i*.*e*., *there exists a constant* $K \in [ 0, 1 )$ *such that* $\varrho ( Tx, Ty ) \leq K \cdot \varrho ( x, y )$ *holds for all* $x, y \in X$. *Then the mapping* *T* *has a unique fixed point*.

Finally, since we want to provide continuous dependence on initial conditions and other data, we need the following inequality named after Gronwall and Bellman.

#### Theorem 3

([43, Theorem 1.3.1])

*Let* $I := [ a, b ]$. *Let* *u*, $f \colon I \longrightarrow [ 0, \infty )$ *be continuous and nonnegative functions*. *Let* $g \colon I \longrightarrow ( 0, \infty )$ *be a continuous*, *positive*, *and nondecreasing function*. *If the inequality*

5 $$ u ( t ) \leq g ( t ) + \int _{a}^{t} f ( s ) \cdot u ( s ) \, \mathrm{d}s $$

*holds for all* $t \in I$, *then we obtain*

6 $$ u ( t ) \leq g ( t ) \cdot \exp \biggl( \int _{a}^{t} f ( s ) \, \mathrm{d}s \biggr) $$

*for all* $t \in I$.

### Continuous problem formulation

At first, let us state the model’s assumptions [16, 17]:

1. The population’s size *N* is fixed over time, i.e., $N ( t ) = N$ for all $t \in [ 0, \infty )$;
2. We divide a population into three homogeneous subgroups, namely susceptible people (S), infectious people (I), and recovered people (R). We can clearly assign every individual to exactly one subgroup. Hence, we obtain
  7 $$ N = S ( t ) + I ( t ) + R ( t ) $$
  for all $t \in [ 0, \infty )$;
3. Additionally, no births and deaths occur;
4. The time-varying transmission rate $\alpha \colon [ 0, \infty ) \longrightarrow [ 0, \infty )$ is Lipschitz continuous and continuously differentiable once, and there exist positive constants $\alpha _{\mathrm{min}} > 0$ and $0 < \alpha _{\mathrm{max}}$ such that $\alpha _{\mathrm{min}} \leq \alpha ( t ) \leq \alpha _{ \mathrm{max}}$ holds for all $t \geq 0$;
5. The time-varying recovery rate $\beta \colon [ 0, \infty ) \longrightarrow [ 0, \infty )$ is Lipschitz continuous and continuously differentiable once, and there are positive constants $\beta _{\mathrm{min}} > 0$ and $0 < \beta _{\mathrm{max}}$ such that $\beta _{\mathrm{min}} \leq \beta ( t ) \leq \beta _{ \mathrm{max}}$ holds for all $t \geq 0$.

We briefly comment on our choice of time-varying transmission rates. The choice of time-dependent transmission rates is possible because countermeasures such as lock-downs, social distancing, or other political actions such as curfews reduce possible contacts between susceptible and infectious people. In addition to that, time-varying recovery rates might also be an interesting choice due to different medical treatments over the course of new epidemics such as COVID-19.

**Figure 1:**
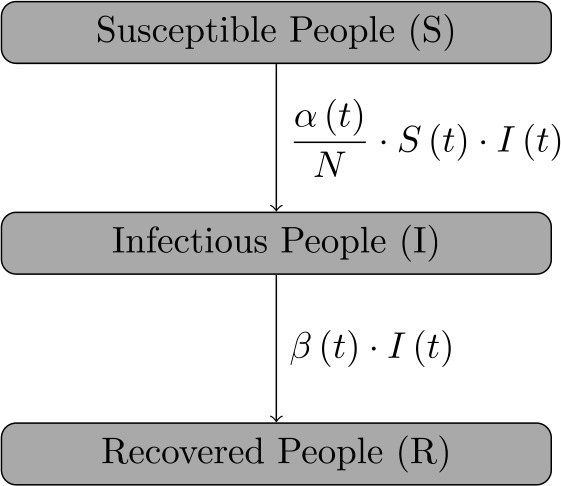
Flowchart of the three different subgroups described by the time-continuous SIR model

Furthermore, we exclude age structures or spatial relationships from our time-continuous model [16, 19]. For abbreviation, we write $f^{\prime } ( t ) := \frac{\mathrm{d} f ( t )}{\mathrm{d}t}$. Our equations of the time-continuous SIR model read as follows:

8 $$ \textstyle\begin{cases} S^{\prime } ( t ) = - \alpha ( t ) \cdot \frac{I ( t ) \cdot S ( t )}{N}, \\ I^{\prime } ( t ) = \alpha ( t ) \cdot \frac{S ( t ) \cdot I ( t )}{N} - \beta ( t ) \cdot I ( t ), \\ R^{\prime } ( t ) = \beta ( t ) \cdot I ( t ) \end{cases} $$

with initial conditions $S ( 0 ) = S_{1} > 0$, $I ( 0 ) = I_{1} > 0$, and $R ( 0 ) = R_{1} \geq 0$. We portray a chart of the flow between the different three subgroups in Fig. 1. Obviously, the equation

$$ N^{\prime } ( t ) = S^{\prime } ( t ) + I^{ \prime } ( t ) + R^{\prime } ( t ) = 0 $$

is valid, and hence the first assumption is fulfilled.

### Nonnegativity and boundedness of solution

Now, we prove boundedness of the solution. For this purpose, we modify ideas from [17] and [44] because we, in contrast to them, consider bounded, time-varying transmission rates $\alpha \colon [ 0, \infty ) \longrightarrow [ 0, \infty )$ and recovery rates $\beta \colon [ 0, \infty ) \longrightarrow [ 0, \infty )$.

#### Lemma 1

*Each solution function of* (8) *is bounded below by zero*.

#### Proof

Here, we split the proof into three parts.

1) We first consider $S^{\prime } ( t ) = - \alpha ( t ) \cdot \frac{I ( t ) \cdot S ( t )}{N}$. Separation of variables leads to

$$ \frac{S^{\prime } ( t )}{S ( t )} = - \alpha ( t ) \cdot \frac{I ( t )}{N}. $$

Integration yields

$$ \ln \biggl( \frac{S ( t )}{S_{1}} \biggr) = - \int _{0}^{t} \alpha ( \tau ) \cdot \frac{I ( \tau )}{N} \, \mathrm{d}\tau , $$

and this implies

$$ S ( t ) = S_{1} \cdot \exp \biggl( - \int _{0}^{t} \alpha ( \tau ) \cdot \frac{I ( \tau )}{N} \, \mathrm{d}\tau \biggr). $$

Hence, it holds $S ( t ) \geq 0$ for all $t \geq 0$.

2) Let us continue with $I^{\prime } ( t ) = \alpha ( t ) \cdot \frac{I ( t ) \cdot S ( t )}{N} - \beta ( t ) \cdot I ( t )$. Separation of variables gives us

$$ \frac{I^{\prime } ( t )}{I ( t )} = \biggl( \alpha ( t ) \cdot \frac{S ( t )}{N} - \beta ( t ) \biggr) $$

and, applying the same argument as in our first step, we conclude

$$ I ( t ) = I_{1} \cdot \exp \biggl( \int _{0}^{t} \biggl\{ \alpha ( \tau ) \cdot \frac{S ( \tau )}{N} - \beta ( t ) \biggr\} \, \mathrm{d}\tau \biggr). $$

This yields $I ( t ) \geq 0$ for all $t \geq 0$.

3) Finally, since $R^{\prime } ( t ) = \beta ( t ) \cdot I ( t )$ holds, we clearly obtain

$$ R ( t ) = R_{1} + \int _{0}^{t} \beta ( t ) \cdot I ( \tau ) \, \mathrm{d}\tau $$

and $R ( t ) \geq 0$ for all $t \geq 0$ is valid.

This completes our proof. □

Since $N = S ( t ) + I ( t ) + R ( t )$ holds by our first assumption, we can finally state our boundedness theorem.

#### Theorem 4

*For all solution functions of* (8), *we have*:

1. $0 \leq S ( t ) \leq N$;
2. $0 \leq I ( t ) \leq N$;
3. $0 \leq R ( t ) \leq N$

*for all* $t \geq 0$.

### Global existence in time

In contrast to many other works, we formulate a theorem regarding global existence in time of (8) based on Theorem 1. For abbreviation, we introduce the supremum norm

$$ \bigl\lVert f ( t ) \bigr\rVert _{\infty } := \sup_{t \in [ a, b ]} \bigl\vert f ( t ) \bigr\vert $$

on an arbitrary time interval $[ a, b ]$ for a continuous function $f \colon [ a, b ] \longrightarrow \mathbb{R}$. A similar definition holds for vector-valued functions. In our case, we consider $[ 0, \infty )$ in general. Now, we show that a possible solution to (8) exists for all times $t \geq 0$.

#### Theorem 5

*The nonlinear first order ordinary differential equation system* (8) *has at least one solution which exists for all* $t \geq 0$.

#### Proof

By defining $\mathbf{z} ( t ) = ( S ( t ), I ( t ), R ( t ) )^{T}$, we can set

9 $$ \mathbf{G} \colon [0, \infty ) \times \mathbb{R}^{3} \longrightarrow \mathbb{R}^{3} ,\qquad \bigl( t, \mathbf{z} ( t ) \bigr) \longrightarrow \begin{pmatrix} - \alpha ( t ) \cdot \frac{I ( t ) \cdot S ( t )}{N} \\ \alpha ( t ) \cdot \frac{S ( t ) \cdot I ( t )}{N} - \beta ( t ) \cdot I ( t ) \\ \beta ( t ) \cdot I ( t ) \end{pmatrix} . $$

Clearly, *G* is Lipschitz continuous. By considering the supremum norm on our Euclidean space and applying the triangle inequality, we get

$$\begin{aligned}& \bigl\lVert \mathbf{G} \bigl( t, \mathbf{z} ( t ) \bigr) \bigr\rVert _{\infty } \\& \quad = \sup_{t \in [ 0, \infty )} \biggl\{ \biggl\vert - \alpha ( t ) \frac{I ( t ) \cdot S ( t )}{N} \biggr\vert , \biggl\vert \alpha ( t ) \frac{S ( t ) \cdot I ( t )}{N} - \beta ( t ) \cdot I ( t ) \biggr\vert , \bigl\vert \beta ( t ) \cdot I ( t ) \bigr\vert \biggr\} \\& \quad \leq \sup_{t \in [ 0, \infty )} \biggl\{ \alpha _{\mathrm{max}} \cdot \biggl\vert \frac{I ( t ) \cdot S ( t )}{N} \biggr\vert , \alpha _{\mathrm{max}} \cdot \biggl\vert \frac{I ( t ) \cdot S ( t )}{N} \biggr\vert + \beta _{\mathrm{max}} \cdot \bigl\vert I ( t ) \bigr\vert , \beta _{\mathrm{max}} \cdot \bigl\vert I ( t ) \bigr\vert \biggr\} \\& \quad \leq \sup_{t \in [ 0, \infty )} \bigl\{ \alpha _{\mathrm{max}} \cdot \bigl\vert S ( t ) \bigr\vert , \alpha _{\mathrm{max}} \cdot \bigl\vert I ( t ) \bigr\vert + \beta _{\mathrm{max}} \cdot \bigl\vert I ( t ) \bigr\vert , \beta _{\mathrm{max}} \cdot \bigl\vert I ( t ) \bigr\vert \bigr\} \\& \quad \leq ( \alpha _{\mathrm{max}} + \beta _{\mathrm{max}} ) \cdot \bigl\lVert \mathbf{z} ( t ) \bigr\rVert _{\infty } \end{aligned}$$

from (9) by the boundedness of our solution functions and the boundedness of our time-varying transmission and recovery rates. Thus, all our assumptions of Theorem 1 are fulfilled and our proof is complete. □

### Global uniqueness in time

We present our proof of global uniqueness in time based on an inductive application of Banach’s fixed point theorem, i.e., that the initial value problem (8) is uniquely solvable for all times $t \geq 0$.

#### Theorem 6

*The nonlinear first order ordinary differential equation system* (8) *has a unique solution that exists for all* $t \geq 0$.

#### Proof

1) Let us first consider the time interval $[ 0, \tau ]$ where we have to choose *τ* accordingly such that Banach’s fixed point theorem is applicable.

2) We need one brief lemma. Let $x_{1}, x_{2}, y_{1}, y_{2} \in \mathbb{R}$ be arbitrary. By zero addition and application of the triangle inequality, we obtain

$$\begin{aligned} \vert x_{1} \cdot y_{1} - x_{2} \cdot y_{2} \vert = & \vert x_{1} \cdot y_{1} - x_{1} \cdot y_{2} + x_{1} \cdot y_{2} - x_{2} \cdot y_{2} \vert \\ \leq & \vert x_{1} \cdot y_{1} - x_{1} \cdot y_{2} \vert + \vert x_{1} \cdot y_{2} - x_{2} \cdot y_{2} \vert \\ = & \vert x_{1} \vert \cdot \vert y_{1} - y_{2} \vert + \vert y_{2} \vert \cdot \vert x_{1} - x_{2} \vert . \end{aligned}$$

3) We assume that *S*, *I*, *R*, *S̃*, *Ĩ*, $\widetilde{R} \colon [ 0, \infty ) \longrightarrow [ 0, \infty )$ are two solutions of (8). At first, it holds

$$\begin{aligned}& \sup_{t \in [ 0, \tau ]} \bigl\vert S ( t ) - \widetilde{S} ( t ) \bigr\vert \\& \quad = \sup_{t \in [ 0, \tau ]} \biggl\vert \int _{0}^{t} - \frac{\alpha ( z )}{N} \cdot S ( z ) \cdot I ( z ) + \frac{\alpha ( z )}{N} \cdot \widetilde{S ( z )} \cdot \widetilde{I ( z )} \, \mathrm{d}z \biggr\vert \\& \quad \leq \sup_{t \in [ 0, \tau ]} \frac{\alpha _{\mathrm{max}}}{N} \cdot \int _{0}^{t} \bigl\vert \widetilde{S ( z )} \cdot \widetilde{I ( z )} - S ( z ) \cdot I ( z ) \bigr\vert \, \mathrm{d}z \\& \quad \leq \sup_{t \in [ 0, \tau ]} \alpha _{ \mathrm{max}} \cdot t \cdot \bigl\{ \bigl\vert I ( t ) - \widetilde{I ( t )} \bigr\vert + \bigl\vert S ( t ) - \widetilde{S ( t )} \bigr\vert \bigr\} \\& \quad \leq 2 \cdot \alpha _{\mathrm{max}} \cdot \tau \cdot \bigl\lVert \mathbf{z} ( t ) - \widetilde{\mathbf{z} ( t )} \bigr\rVert _{\infty } \end{aligned}$$

by our inequality of the second step.

4) Secondly, we obtain

$$\begin{aligned}& \sup_{t \in [ 0, \tau ]} \bigl\vert I ( t ) - \widetilde{I ( t )} \bigr\vert \\& \quad = \sup_{t \in [ 0, \tau ]} \biggl\vert \int _{0}^{t} \frac{\alpha ( z )}{N} \cdot \bigl\{ I ( z ) \cdot S ( z ) - \widetilde{I ( z )} \cdot \widetilde{S ( z )} \bigr\} + \beta ( z ) \cdot \bigl\{ I ( z ) - \widetilde{I ( z )} \bigr\} \, \mathrm{d}z \biggr\vert \\& \quad \leq \sup_{t \in [ 0, \tau ]} \frac{\alpha _{\mathrm{max}}}{N} \cdot \int _{0}^{t} \bigl\vert I ( z ) \cdot S ( z ) - \widetilde{I ( z )} \cdot \widetilde{S ( z )} \bigr\vert \, \mathrm{d}z + \sup_{t \in [ 0, \tau ]} \beta _{\mathrm{max}} \cdot \int _{0}^{t} \bigl\vert I ( z ) - \widetilde{I ( z )} \bigr\vert \, \mathrm{d}z \\& \quad \leq \sup_{t \in [ 0, \tau ]} \alpha _{ \mathrm{max}} \cdot t \cdot \bigl\{ \bigl\vert I ( t ) - \widetilde{I ( t )} \bigr\vert + \bigl\vert S ( t ) - \widetilde{S ( t )} \bigr\vert \bigr\} + \sup_{t \in [ 0, \tau ]} \beta _{\mathrm{max}} \cdot t \cdot \bigl\vert I ( t ) - \widetilde{I ( t )} \bigr\vert \\& \quad \leq ( 2 \cdot \alpha _{\mathrm{max}} + \beta _{\mathrm{max}} ) \cdot \tau \cdot \bigl\lVert \mathbf{z} ( t ) - \widetilde{\mathbf{z} ( t )} \bigr\rVert _{\infty } \end{aligned}$$

by our inequality of the second step and application of the triangle inequality.

5) Furthermore, we conclude that

$$\begin{aligned}& \sup_{t \in [ 0, \tau ]} \bigl\vert R ( t ) - \widetilde{R ( t )} \bigr\vert \\& \quad = \sup_{t \in [ 0, \tau ]} \biggl\vert \int _{0}^{t} \beta ( z ) \cdot \bigl\{ I ( z ) - \widetilde{I ( z )} \bigr\} \, \mathrm{d}z \biggr\vert \\& \quad \leq \beta _{\mathrm{max}} \cdot \tau \cdot \bigl\lVert \mathbf{z} ( t ) - \widetilde{\mathbf{z} ( t )} \bigr\rVert _{\infty } \end{aligned}$$

holds.

6) Summarizing the previous steps, we obtain

$$ \bigl\lVert \mathbf{z} ( t ) - \widetilde{\mathbf{z} ( t )} \bigr\rVert _{\infty } \leq ( 2 \cdot \alpha _{\mathrm{max}} + \beta _{\mathrm{max}} ) \cdot \tau \cdot \bigl\lVert \mathbf{z} ( t ) - \widetilde{ \mathbf{z} ( t )} \bigr\rVert _{\infty }. $$

If we choose $\tau := \frac{1}{2 \cdot ( 2 \cdot \alpha _{\mathrm{max}} + \beta _{\mathrm{max}} ) }$, this implies

$$ \bigl\lVert \mathbf{z} ( t ) - \widetilde{\mathbf{z} ( t )} \bigr\rVert _{\infty } \leq \frac{1}{2} \cdot \bigl\lVert \mathbf{z} ( t ) - \widetilde{\mathbf{z} ( t )} \bigr\rVert _{\infty }, $$

and hence we conclude the uniqueness of solution on the time interval $[ 0, \tau ]$.

7) Inductively, we see that we can derive this contraction property on all time intervals $[ k \cdot \tau , ( k + 1 ) \cdot \tau ]$ for all $k \in \mathbb{N}_{0}$ by choosing $k \cdot \tau $ as our starting point and for our initial conditions. Henceforth, our proof of global uniqueness in time is complete. □

### Continuous dependence on initial conditions and time-varying rates

Here, we consider the perturbed initial value problems

10 $$ \textstyle\begin{cases} S_{a}^{\prime } ( t ) = - \alpha _{a} ( t ) \cdot \frac{I_{a} ( t ) \cdot S_{a} ( t )}{N}, \\ I_{a}^{\prime } ( t ) = \alpha _{a} ( t ) \cdot \frac{S_{a} ( t ) \cdot I_{a} ( t )}{N} - \beta _{a} ( t ) \cdot I_{a} ( t ), \\ R_{a}^{\prime } ( t ) = \beta _{a} ( t ) \cdot I_{a} ( t ) \end{cases} $$

with initial conditions $S_{a} ( 0 ) = S_{a, 1} > 0$, $I_{a} ( 0 ) = I_{a, 1} > 0$, $R_{a} ( 0 ) = R_{a, 1} \geq 0$ and

11 $$ \textstyle\begin{cases} S_{b}^{\prime } ( t ) = - \alpha _{b} ( t ) \cdot \frac{I_{b} ( t ) \cdot S_{b} ( t )}{N}, \\ I_{b}^{\prime } ( t ) = \alpha _{b} ( t ) \cdot \frac{S_{b} ( t ) \cdot I_{b} ( t )}{N} - \beta _{b} ( t ) \cdot I_{b} ( t ), \\ R_{b}^{\prime } ( t ) = \beta _{b} ( t ) \cdot I_{b} ( t ) \end{cases} $$

with initial conditions $S_{b} ( 0 ) = S_{b, 1} > 0$, $I_{b} ( 0 ) = I_{b, 1} > 0$, $R_{b} ( 0 ) = R_{b, 1} \geq 0$, where $\alpha _{a}$, $\alpha _{b}$, $\beta _{a}$, $\beta _{b} \colon [ 0, \infty ) \longrightarrow [ 0, \infty )$ are different time-varying transmission and recovery rates. Now, we prove that small perturbations in initial conditions or small differences in time-varying transmission or recovery rates lead to small differences in the solutions on short time intervals $[ 0, T ]$. This fact is summarized in the following theorem.

#### Theorem 7

*Let* $\mathbf{z}_{a} ( t ) = ( S_{a} ( t ), I_{a} ( t ), R_{a} ( t ) )^{T}$ *and* $\mathbf{z}_{b} ( t ) = ( S_{b} ( t ), I_{b} ( t ), R_{b} ( t ) )^{T}$ *be the solutions of* (10) *and* (11). *Define the function*

$$\begin{aligned} g ( t ) := & \bigl\lVert \mathbf{z}_{a} ( 0 ) - \mathbf{z}_{b} ( 0 ) \bigr\rVert _{\infty } + N_{a} \cdot t \cdot \bigl\lVert \alpha _{a} ( t ) - \alpha _{b} ( t ) \bigr\rVert _{\infty } \\ & {}+ \frac{N_{b}}{N_{a}} \cdot t \cdot \max \{ \alpha _{ \mathrm{max,a}}; \alpha _{\mathrm{max,b}} \} \cdot \vert N_{a} - N_{b} \vert + N_{a} \cdot t \cdot \bigl\lVert \beta _{a} ( t ) - \beta _{b} ( t ) \bigr\rVert _{\infty } \end{aligned}$$

*and the constant*

$$ K_{\mathrm{GB}} := \biggl\{ \max \{ \alpha _{\mathrm{max,a}}; \alpha _{\mathrm{max,b}} \} \cdot \biggl( 1 + \frac{N_{b}}{N_{a}} \biggr) + \max \{ \beta _{\mathrm{max,a}}; \beta _{\mathrm{max,b}} \} \biggr\} . $$

*We see that*

12 $$ \bigl\lVert \mathbf{z}_{a} ( t ) - \mathbf{z}_{b} ( t ) \bigr\rVert _{\infty } \leq g ( t ) \cdot \exp ( K_{\mathrm{GB}} \cdot t ) $$

*holds for arbitrary* $t \in [ 0, T ]$ *with given* $T \geq 0$.

#### Proof

1) Let us first mention that we often use the inequality

$$ \vert x_{1} \cdot y_{1} - x_{2} \cdot y_{2} \vert \leq \vert x_{1} \vert \cdot \vert y_{1} - y_{2} \vert + \vert y_{2} \vert \cdot \vert x_{1} - x_{2} \vert $$

for arbitrary $x_{1}, x_{2}, y_{1}, y_{2} \in \mathbb{R}$ as proven in Theorem 6. Additionally, we see that

$$ N_{a} = S_{a} ( 0 ) + I_{a} ( 0 ) + R_{a} ( 0 ) \quad \text{and}\quad N_{b} = S_{b} ( 0 ) + I_{b} ( 0 ) + R_{b} ( 0 ) $$

hold for all $t \in [ 0, T ]$.

2) At first, we obtain the inequality

$$\begin{aligned}& \bigl\vert S_{a} ( t ) - S_{b} ( t ) \bigr\vert \\& \quad \leq \bigl\vert S_{a} ( 0 ) - S_{b} ( 0 ) \bigr\vert + \int _{0}^{t} \biggl\vert \frac{\alpha _{a} ( \tau )}{N_{a}} \cdot I_{a} ( \tau ) \cdot S_{a} ( \tau ) - \frac{\alpha _{b} ( \tau )}{N_{b}} \cdot I_{b} ( \tau ) \cdot S_{b} ( \tau ) \biggr\vert \, \mathrm{d}\tau \\& \quad \leq \bigl\vert S_{a} ( 0 ) - S_{b} ( 0 ) \bigr\vert + \int _{0}^{t} \biggl\vert \frac{\alpha _{a} ( \tau )}{N_{a}} \cdot I_{a} ( \tau ) \cdot S_{a} ( \tau ) - \frac{\alpha _{b} ( \tau )}{N_{a}} \cdot I_{a} ( \tau ) \cdot S_{a} ( \tau ) \biggr\vert \, \mathrm{d}\tau \\& \qquad {}+ \int _{0}^{t} \biggl\vert \frac{\alpha _{b} ( \tau )}{N_{a}} \cdot I_{a} ( \tau ) \cdot S_{a} ( \tau ) - \frac{\alpha _{b} ( \tau )}{N_{a}} \cdot I_{a} ( \tau ) \cdot S_{b} ( \tau ) \biggr\vert \, \mathrm{d}\tau \\& \qquad {}+ \int _{0}^{t} \biggl\vert \frac{\alpha _{b} ( \tau )}{N_{a}} \cdot I_{a} ( \tau ) \cdot S_{b} ( \tau ) - \frac{\alpha _{b} ( \tau )}{N_{b}} \cdot I_{b} ( \tau ) \cdot S_{b} ( \tau ) \biggr\vert \, \mathrm{d}\tau \\& \quad \leq \bigl\vert S_{a} ( 0 ) - S_{b} ( 0 ) \bigr\vert + N_{a} \cdot t \cdot \bigl\lVert \alpha _{a} ( t ) - \alpha _{b} ( t ) \bigr\rVert _{\infty } \\& \qquad {}+ \max \{ \alpha _{\mathrm{max,a}}; \alpha _{ \mathrm{max,b}} \} \cdot \int _{0}^{t} \bigl\vert S_{a} ( \tau ) - S_{b} ( \tau ) \bigr\vert \, \mathrm{d}\tau \\& \qquad {}+ \max \{ \alpha _{\mathrm{max,a}}; \alpha _{ \mathrm{max,b}} \} \cdot N_{b} \cdot \int _{0}^{t} \biggl\vert \frac{1}{N_{a}} \cdot I_{a} ( \tau ) - \frac{1}{N_{b}} \cdot I_{b} ( \tau ) \biggr\vert \, \mathrm{d}\tau \\& \quad \leq \bigl\vert S_{a} ( 0 ) - S_{b} ( 0 ) \bigr\vert + N_{a} \cdot t \cdot \bigl\lVert \alpha _{a} ( t ) - \alpha _{b} ( t ) \bigr\rVert _{\infty } \\& \qquad {}+ \max \{ \alpha _{\mathrm{max,a}}; \alpha _{ \mathrm{max,b}} \} \cdot \int _{0}^{t} \bigl\vert S_{a} ( \tau ) - S_{b} ( \tau ) \bigr\vert \, \mathrm{d}\tau \\& \qquad {}+ \max \{ \alpha _{\mathrm{max,a}}; \alpha _{ \mathrm{max,b}} \} \cdot N_{b} \cdot \int _{0}^{t} \biggl\{ \frac{1}{N_{a}} \cdot \bigl\vert I_{a} ( \tau ) - I_{b} ( \tau ) \bigr\vert + N_{b} \cdot \biggl\vert \frac{1}{N_{a}} - \frac{1}{N_{b}} \biggr\vert \biggr\} \, \mathrm{d}\tau \\& \quad \leq \bigl\vert S_{a} ( 0 ) - S_{b} ( 0 ) \bigr\vert + N_{a} \cdot t \cdot \bigl\lVert \alpha _{a} ( t ) - \alpha _{b} ( t ) \bigr\rVert _{\infty } \\& \qquad {}+ \max \{ \alpha _{\mathrm{max,a}}; \alpha _{ \mathrm{max,b}} \} \cdot \int _{0}^{t} \bigl\vert S_{a} ( \tau ) - S_{b} ( \tau ) \bigr\vert \, \mathrm{d}\tau \\& \qquad {}+ \max \{ \alpha _{\mathrm{max,a}}; \alpha _{ \mathrm{max,b}} \} \cdot \frac{N_{b}}{N_{a}} \cdot \int _{0}^{t} \bigl\vert I_{a} ( \tau ) - I_{b} ( \tau ) \bigr\vert \, \mathrm{d}\tau \\& \qquad {}+ \frac{N_{b}}{N_{a}} \cdot t \cdot \max \{ \alpha _{ \mathrm{max,a}}; \alpha _{\mathrm{max,b}} \} \cdot \vert N_{a} - N_{b} \vert \end{aligned}$$

for arbitrary $t \in [ 0, T ]$ by application of the triangle inequality, boundedness of our all functions, and the inequality of our first step.

3) Secondly, we have to estimate $\vert I_{a} ( t ) - I_{b} ( t ) \vert $. We see that

$$\begin{aligned}& \bigl\vert I_{a} ( t ) - I_{b} ( t ) \bigr\vert \\& \quad \leq \bigl\vert I_{a} ( 0 ) - I_{b} ( 0 ) \bigr\vert + \underbrace{ \int _{0}^{t} \biggl\vert \frac{\alpha _{a} ( \tau )}{N_{a}} \cdot I_{a} ( \tau ) \cdot S_{a} ( \tau ) - \frac{\alpha _{b} ( \tau )}{N_{b}} \cdot I_{b} ( \tau ) \cdot S_{b} ( \tau ) \biggr\vert \, \mathrm{d}\tau }_{:= I} \\& \qquad {}+ \underbrace{ \int _{0}^{t} \bigl\vert \beta _{a} ( \tau ) \cdot I_{a} ( \tau ) - \beta _{b} ( \tau ) \cdot I_{b} ( \tau ) \bigr\vert \, \mathrm{d}\tau }_{:= \mathit{II}} \end{aligned}$$

holds for arbitrary $t \in [ 0, T ]$. The summand *I* can be estimated in the previous step. This yields

$$\begin{aligned} I \leq & N_{a} \cdot t \cdot \bigl\lVert \alpha _{a} ( t ) - \alpha _{b} ( t ) \bigr\rVert _{\infty } + \max \{ \alpha _{\mathrm{max,a}}; \alpha _{\mathrm{max,b}} \} \cdot \int _{0}^{t} \bigl\vert S_{a} ( \tau ) - S_{b} ( \tau ) \bigr\vert \, \mathrm{d}\tau \\ & {}+ \max \{ \alpha _{\mathrm{max,a}}; \alpha _{ \mathrm{max,b}} \} \cdot \frac{N_{b}}{N_{a}} \cdot \int _{0}^{t} \bigl\vert I_{a} ( \tau ) - I_{b} ( \tau ) \bigr\vert \, \mathrm{d}\tau \\ & {}+ \frac{N_{b}}{N_{a}} \cdot t \cdot \max \{ \alpha _{ \mathrm{max,a}}; \alpha _{\mathrm{max,b}} \} \cdot \vert N_{a} - N_{b} \vert \end{aligned}$$

for arbitrary $t \in [ 0, T ]$. For the third summand *II*, we observe that

$$\begin{aligned} \mathit{II} \leq & \int _{0}^{t} \bigl\vert \beta _{a} ( \tau ) \cdot I_{a} ( \tau ) - \beta _{b} ( \tau ) \cdot I_{b} ( \tau ) \bigr\vert \, \mathrm{d}\tau \\ \leq & \int _{0}^{t} \bigl\vert \beta _{a} ( \tau ) \cdot I_{a} ( \tau ) - \beta _{b} ( \tau ) \cdot I_{a} ( \tau ) \bigr\vert \, \mathrm{d}\tau + \int _{0}^{t} \bigl\vert \beta _{b} ( \tau ) \cdot I_{a} ( \tau ) - \beta _{b} ( \tau ) \cdot I_{b} ( \tau ) \bigr\vert \, \mathrm{d}\tau \\ \leq & N_{a} \cdot t \cdot \bigl\lVert \beta _{a} ( t ) - \beta _{b} ( t ) \bigr\rVert _{\infty } + \max \{ \beta _{ \mathrm{max,a}}; \beta _{\mathrm{max,b}} \} \cdot \int _{0}^{t} \bigl\vert I_{a} ( \tau ) - I_{b} ( \tau ) \bigr\vert \, \mathrm{d}\tau \end{aligned}$$

is valid for arbitrary *t*. Summarizing these results, we obtain

$$\begin{aligned}& \bigl\vert I_{a} ( t ) - I_{b} ( t ) \bigr\vert \\& \quad \leq \bigl\vert I_{a} ( 0 ) - I_{b} ( 0 ) \bigr\vert + N_{a} \cdot t \cdot \bigl\lVert \alpha _{a} ( t ) - \alpha _{b} ( t ) \bigr\rVert _{\infty } \\& \qquad {}+ \frac{N_{b}}{N_{a}} \cdot t \cdot \max \{ \alpha _{ \mathrm{max,a}}; \alpha _{\mathrm{max,b}} \} \cdot \vert N_{a} - N_{b} \vert + N_{a} \cdot t \cdot \bigl\lVert \beta _{a} ( t ) - \beta _{b} ( t ) \bigr\rVert _{\infty } \\& \qquad {}+ \max \{ \alpha _{\mathrm{max,a}}; \alpha _{ \mathrm{max,b}} \} \cdot \int _{0}^{t} \bigl\vert S_{a} ( \tau ) - S_{b} ( \tau ) \bigr\vert \, \mathrm{d}\tau \\& \qquad {}+ \max \{ \alpha _{\mathrm{max,a}}; \alpha _{ \mathrm{max,b}} \} \cdot \frac{N_{b}}{N_{a}} \cdot \int _{0}^{t} \bigl\vert I_{a} ( \tau ) - I_{b} ( \tau ) \bigr\vert \, \mathrm{d}\tau \\& \qquad {}+ \max \{ \beta _{\mathrm{max,a}}; \beta _{ \mathrm{max,b}} \} \cdot \int _{0}^{t} \bigl\vert I_{a} ( \tau ) - I_{b} ( \tau ) \bigr\vert \, \mathrm{d}\tau \end{aligned}$$

for arbitrary $t \in [ 0, T ]$.

4) Now, we must estimate $\vert R_{a} ( t ) - R_{b} ( t ) \vert $. It holds

$$\begin{aligned}& \bigl\vert R_{a} ( t ) - R_{b} ( t ) \bigr\vert \\& \quad \leq \bigl\vert R_{a} ( 0 ) - R_{b} ( 0 ) \bigr\vert + \int _{0}^{t} \bigl\vert \beta _{a} ( \tau ) \cdot I_{a} ( \tau ) - \beta _{b} ( \tau ) \cdot I_{b} ( \tau ) \bigr\vert \, \mathrm{d}\tau \\& \quad \leq \bigl\vert R_{a} ( 0 ) - R_{b} ( 0 ) \bigr\vert + \int _{0}^{t} \bigl\vert \beta _{a} ( \tau ) \cdot I_{a} ( \tau ) - \beta _{b} ( \tau ) \cdot I_{a} ( \tau ) \bigr\vert \, \mathrm{d}\tau \\& \qquad {}+ \int _{0}^{t} \bigl\vert \beta _{b} ( \tau ) \cdot I_{a} ( \tau ) - \beta _{b} ( \tau ) \cdot I_{b} ( \tau ) \bigr\vert \, \mathrm{d}\tau \\& \quad \leq \bigl\vert R_{a} ( 0 ) - R_{b} ( 0 ) \bigr\vert + N_{a} \cdot t \cdot \bigl\lVert \beta _{a} ( t ) - \beta _{b} ( t ) \bigr\rVert _{\infty } \\& \qquad {}+ \max \{ \beta _{\mathrm{max,a}}; \beta _{ \mathrm{max,b}} \} \cdot \int _{0}^{t} \bigl\vert I_{a} ( \tau ) - I_{b} ( \tau ) \bigr\vert \, \mathrm{d}\tau \end{aligned}$$

for arbitrary $t \in [ 0, T ]$.

5) Finally, we obtain

$$\begin{aligned}& \bigl\lVert \mathbf{z}_{a} ( t ) - \mathbf{z}_{b} ( t ) \bigr\rVert _{\infty } \\& \quad \leq \bigl\lVert \mathbf{z}_{a} ( 0 ) - \mathbf{z}_{b} ( 0 ) \bigr\rVert _{\infty } + N_{a} \cdot t \cdot \bigl\lVert \alpha _{a} ( t ) - \alpha _{b} ( t ) \bigr\rVert _{\infty } \\& \qquad {}+ \frac{N_{b}}{N_{a}} \cdot t \cdot \max \{ \alpha _{ \mathrm{max,a}}; \alpha _{\mathrm{max,b}} \} \cdot \vert N_{a} - N_{b} \vert + N_{a} \cdot t \cdot \bigl\lVert \beta _{a} ( t ) - \beta _{b} ( t ) \bigr\rVert _{\infty } \\& \qquad {}+ \max \{ \alpha _{\mathrm{max,a}}; \alpha _{ \mathrm{max,b}} \} \cdot \int _{0}^{t} \bigl\lVert \mathbf{z}_{a} ( \tau ) - \mathbf{z}_{b} ( \tau ) \bigr\rVert _{\infty } \, \mathrm{d}\tau \\& \qquad {}+ \max \{ \alpha _{\mathrm{max,a}}; \alpha _{ \mathrm{max,b}} \} \cdot \frac{N_{b}}{N_{a}} \cdot \int _{0}^{t} \bigl\lVert \mathbf{z}_{a} ( \tau ) - \mathbf{z}_{b} ( \tau ) \bigr\rVert _{\infty } \, \mathrm{d} \tau \\& \qquad {}+ \max \{ \beta _{\mathrm{max,a}}; \beta _{ \mathrm{max,b}} \} \cdot \int _{0}^{t} \bigl\lVert \mathbf{z}_{a} ( \tau ) - \mathbf{z}_{b} ( \tau ) \bigr\rVert _{\infty } \, \mathrm{d}\tau \end{aligned}$$

for arbitrary $t \in [ 0, T ]$. This implies

$$\begin{aligned}& \bigl\lVert \mathbf{z}_{a} ( t ) - \mathbf{z}_{b} ( t ) \bigr\rVert _{\infty } \\& \quad \leq \bigl\lVert \mathbf{z}_{a} ( 0 ) - \mathbf{z}_{b} ( 0 ) \bigr\rVert _{\infty } + N_{a} \cdot t \cdot \bigl\lVert \alpha _{a} ( t ) - \alpha _{b} ( t ) \bigr\rVert _{\infty } \\& \qquad {}+ \frac{N_{b}}{N_{a}} \cdot t \cdot \max \{ \alpha _{ \mathrm{max,a}}; \alpha _{\mathrm{max,b}} \} \cdot \vert N_{a} - N_{b} \vert + N_{a} \cdot t \cdot \bigl\lVert \beta _{a} ( t ) - \beta _{b} ( t ) \bigr\rVert _{\infty } \\& \qquad {}+ \biggl\{ \max \{ \alpha _{\mathrm{max,a}}; \alpha _{ \mathrm{max,b}} \} \cdot \int _{0}^{t} \bigl\lVert \mathbf{z}_{a} ( \tau ) - \mathbf{z}_{b} ( \tau ) \bigr\rVert _{\infty } \, \mathrm{d}\tau \\& \qquad {}+ \max \{ \alpha _{\mathrm{max,a}}; \alpha _{ \mathrm{max,b}} \} \cdot \frac{N_{b}}{N_{a}} \cdot \int _{0}^{t} \bigl\lVert \mathbf{z}_{a} ( \tau ) - \mathbf{z}_{b} ( \tau ) \bigr\rVert _{\infty } \, \mathrm{d} \tau \\& \qquad {}+ \max \{ \beta _{\mathrm{max,a}}; \beta _{ \mathrm{max,b}} \} \cdot \int _{0}^{t} \bigl\lVert \mathbf{z}_{a} ( \tau ) - \mathbf{z}_{b} ( \tau ) \bigr\rVert _{\infty } \, \mathrm{d}\tau \biggr\} . \end{aligned}$$

6) Define the functions

$$\begin{aligned}& u ( t ) := \bigl\lVert \mathbf{z}_{a} ( t ) - \mathbf{z}_{b} ( t ) \bigr\rVert _{\infty }, \\& \begin{aligned} g ( t ) := {}& \bigl\lVert \mathbf{z}_{a} ( 0 ) - \mathbf{z}_{b} ( 0 ) \bigr\rVert _{\infty } + N_{a} \cdot t \cdot \bigl\lVert \alpha _{a} ( t ) - \alpha _{b} ( t ) \bigr\rVert _{\infty } \\ &{}+ \frac{N_{b}}{N_{a}} \cdot t \cdot \max \{ \alpha _{ \mathrm{max,a}}; \alpha _{\mathrm{max,b}} \} \cdot \vert N_{a} - N_{b} \vert + N_{a} \cdot t \cdot \bigl\lVert \beta _{a} ( t ) - \beta _{b} ( t ) \bigr\rVert _{\infty }, \end{aligned} \\& f ( t ) := \biggl\{ \max \{ \alpha _{ \mathrm{max,a}}; \alpha _{\mathrm{max,b}} \} \cdot \biggl( 1 + \frac{N_{b}}{N_{a}} \biggr) + \max \{ \beta _{\mathrm{max,a}}; \beta _{\mathrm{max,b}} \} \biggr\} =: K_{\mathrm{GB}}. \end{aligned}$$

Since all the assumptions of Theorem 3 are fulfilled, we see that

$$ \bigl\lVert \mathbf{z}_{a} ( t ) - \mathbf{z}_{b} ( t ) \bigr\rVert _{\infty } \leq g ( t ) \cdot \exp ( K_{\mathrm{GB}} \cdot t ) $$

holds for arbitrary $t \in [ 0, T ]$, which finishes our proof. □

### Monotonicity properties and long-time behavior

We now investigate the long-time behavior of solution, some monotonicity properties and summarize our results in the following theorem.

#### Theorem 8

*We get*:

1. *S* *is monotonically decreasing*, *and there exists a number* $S^{\star } \geq 0$ *such that* $\lim_{t \to \infty } S ( t ) = S^{\star }$. *It even holds* $S^{\star } > 0$;
2. *R* *is monotonically increasing*, *and there exists a number* $R^{\star } \geq 0$ *such that* $\lim_{t \to \infty } R ( t ) = R^{\star }$;
3. *I* *is Lebesgue*-*integrable on* $[ 0, \infty )$ *and* $\lim_{t \to \infty } I ( t ) = 0$

*for all solution functions of* (8).

#### Proof

1) Since $S^{\prime } ( t ) \leq 0$ for all $t \geq 0$ and $0 \leq S ( t ) \leq S_{0}$ by Theorem 4, $S \colon [ 0, \infty ) \longrightarrow [ 0, \infty )$ is monotonically decreasing and bounded below by zero. This implies the existence of $S^{\star } \geq 0$ such that $\lim_{t \to \infty } S ( t ) = S^{\star }$. Additionally, by considering $\frac{S^{\prime } ( t )}{R^{\prime } ( t )}$ for $t > 0$, we obtain

$$ \frac{S^{\prime } ( t )}{R^{\prime } ( t )} = - \frac{\alpha ( t ) \cdot S ( t )}{\beta ( t ) \cdot N} \geq - \frac{\alpha _{\mathrm{max}}}{\beta _{\mathrm{min}}} \cdot \frac{S ( t )}{N}, $$

and separation of variables implies

$$ \frac{S^{\prime } ( t )}{S ( t )} \geq - \frac{\alpha _{\mathrm{max}}}{\beta _{\mathrm{min}} \cdot N} \cdot R^{ \prime } ( t ). $$

Integration yields

$$ S ( t ) \geq S ( 0 ) \cdot \exp \biggl( - \frac{\alpha _{\mathrm{max}}}{\beta _{\mathrm{min}} \cdot N} \cdot \bigl( R ( t ) - R ( 0 ) \bigr) \biggr) \geq S ( 0 ) \cdot \exp \biggl( - \frac{\alpha _{\mathrm{max}}}{\beta _{\mathrm{min}}} \biggr) > 0 $$

for all $t \geq 0$. Hence, it holds $S^{\star } > 0$.

2) Since $R^{\prime } ( t ) \geq 0$ for all $t \geq 0$ and $0 \leq R ( t ) \leq N$ is true by application of Theorem 4, $R \colon [ 0, \infty ) \longrightarrow [ 0, \infty )$ is monotonically increasing and bounded above by *N*. This yields the existence of $R^{\star } \geq 0$ such that $\lim_{t \to \infty } R ( t ) = R^{\star }$ holds.

3) Since $S^{\prime } ( t ) = - \alpha ( t ) \cdot \frac{I ( t ) \cdot S ( t )}{N}$ holds, integration yields

$$\begin{aligned} S_{1} - S^{\star } = & \int _{0}^{\infty } \frac{\alpha ( \tau )}{N} \cdot S ( \tau ) \cdot I ( \tau ) \, \mathrm{d}\tau \\ \geq & \frac{\alpha _{\mathrm{min}} \cdot S^{\star }}{N} \cdot \int _{0}^{t} I ( \tau ) \, \mathrm{d}\tau \end{aligned}$$

because all functions *α*, *S*, $I \colon [ 0, \infty ) \longrightarrow [ 0, \infty )$ are bounded and nonnegative. Therefore, we obtain that *I* is Lebesgue-integrable on $[ 0, \infty )$ and $\lim_{t \to \infty } I ( t ) = 0$. This finishes our proof. □

### Calculation of the time-continuous basic reproduction number

In our nonautonomous SIR model, the time-dependent basic reproduction number can be defined by

13 $$ \mathcal{R}_{0} ( t ) := \frac{\alpha ( t )}{\beta ( t )}, $$

which is similar to the constant case [17, 45, 46].

#### Lemma 2

*Equation* (13) *is well defined*.

#### Proof

We observe that

$$ 0 < \frac{\alpha _{\mathrm{min}}}{\beta _{\mathrm{max}}} \leq \frac{\alpha ( t )}{\beta ( t )} =: \mathcal{R}_{0} ( t ) := \frac{\alpha ( t )}{\beta ( t )} \leq \frac{\alpha _{\mathrm{max}}}{\beta _{\mathrm{min}}} $$

is valid for all $t \geq 0$. This proves our claim. □

## Time-discrete implicit SIR model

In this section, we examine time-discrete versions of the given time-continuous SIR model (8). Let us assume that our time interval $[ 0, T ]$ can be divided by a strictly increasing sequence $\{ t_{j} \} _{j = 1}^{M}$ for $M \in \mathbb{N}$ with $t_{1} = 0 $ and $t_{M} = T$. For abbreviation, we write $f ( t_{j} ) := f_{j}$ for all $j \in \{ 1, \ldots , M \} $ and an arbitrary time-dependent function *f*.

### Discussion of formulations

Here, we only state a fully explicit scheme

14 $$ \textstyle\begin{cases} \frac{S_{j + 1} - S_{j}}{t_{j + 1} - t_{j}} = - \alpha _{j + 1} \cdot \frac{I_{j} \cdot S_{j}}{N}, \\ \frac{I_{j + 1} - I_{j}}{t_{j + 1} - t_{j}} = \alpha _{j + 1} \cdot \frac{I_{j} \cdot S_{j}}{N} - \beta _{j + 1} \cdot I_{j}, \\ \frac{R_{j + 1} - R_{j}}{t_{j + 1} - t_{j}} = \beta _{j + 1} \cdot I_{j} \end{cases} $$

and a fully implicit scheme

15 $$ \textstyle\begin{cases} \frac{S_{j + 1} - S_{j}}{t_{j + 1} - t_{j}} = - \alpha _{j + 1} \cdot \frac{I_{j + 1} \cdot S_{j + 1}}{N}, \\ \frac{I_{j + 1} - I_{j}}{t_{j + 1} - t_{j}} = \alpha _{j + 1} \cdot \frac{I_{j + 1} \cdot S_{j + 1}}{N} - \beta _{j + 1} \cdot I_{j + 1}, \\ \frac{R_{j + 1} - R_{j}}{t_{j + 1} - t_{j}} = \beta _{j + 1} \cdot I_{j + 1} \end{cases} $$

of the time-continuous SIR model (8) for all $j \in \{ 1, \ldots , M - 1 \} $. Both formulations fulfill

16 $$ N = S_{j + 1} + I_{j + 1} + R_{j + 1} = S_{j} + I_{j} + R_{j} $$

for all $j \in \{ 1, \ldots , M - 1 \} $. However, the fully explicit scheme (14) simply reduces to a linear system, while the fully implicit scheme (15) maintains the nonlinear structure of the continuous problem formulation (8). For this reason, we investigate this fully implicit scheme in the following.

### Time-discrete implicit problem formulation

We assume that $0 < \alpha _{\mathrm{min}} \leq a_{j} \leq \alpha _{\mathrm{max}} < 1$ and $0 < \beta _{\mathrm{min}} \leq \beta _{j} < \beta _{\mathrm{max}} \leq 1$ are given for all $j \in \{ 1, \ldots , M \} $ and that $0 < t_{j + 1} - t_{j} \leq 1$ for all $j \in \{ 1, \ldots , M - 1 \} $ and that $S_{1} > 0$, $I_{1} > 0$, and $R_{1} \geq 0$ are given. As later observed in our numerical examples, these assumptions are fulfilled in epidemiological data of the spread of COVID-19. An implicit solution scheme of (15) reads as follows:

17 $$ \textstyle\begin{cases} S_{j + 1} = \frac{S_{j}}{1 + \alpha _{j + 1} \cdot ( t_{j + 1} - t_{j} ) \cdot \frac{I_{j + 1}}{N}}, \\ I_{j + 1} = \frac{I_{j}}{1 + \beta _{j + 1} \cdot ( t_{j + 1} - t_{j} ) - \alpha _{j + 1} \cdot ( t_{j + 1} - t_{j} ) \cdot \frac{S_{j + 1}}{N}}, \\ R_{j + 1} = R_{j} + \beta _{j + 1} \cdot ( t_{j + 1} - t_{j} ) \cdot I_{j + 1} \end{cases} $$

for all $j \in \{ 1, \ldots , M - 1 \} $. Now, we are able to obtain an appropriate solution scheme from (17) which even implies unique solvability for all $j \in \{ 1, \ldots , M - 1 \} $ under the assumption that $S_{j} > 0$, $I_{j} > 0$, and $R_{j} \geq 0$ for all $j \in \{ 1, \ldots , M \} $.

### Unique solvability

Our main ingredient is the equation

18 $$ I_{j + 1} = \frac{I_{j}}{1 + \beta _{j + 1} \cdot ( t_{j + 1} - t_{j} ) - \alpha _{j + 1} \cdot ( t_{j + 1} - t_{j} ) \cdot \frac{S_{j + 1}}{N}} $$

from (17). Plugging

$$ S_{j + 1} = \frac{S_{j}}{1 + \alpha _{j + 1} \cdot ( t_{j + 1} - t_{j} ) \cdot \frac{I_{j + 1}}{N}} $$

into (18) and writing $\Delta _{j + 1} = ( t_{j + 1} - t_{j} )$ yields

19 $$ I_{j + 1} = \frac{ ( N + \alpha _{j + 1} \cdot \Delta _{j + 1} \cdot I_{j + 1} ) \cdot I_{j}}{ ( 1 + \beta _{j + 1} \cdot \Delta _{j + 1} ) \cdot ( N + \alpha _{j + 1} \cdot \Delta _{j + 1} \cdot I_{j + 1} ) - \alpha _{j + 1} \cdot \Delta _{j + 1} \cdot S_{j}} $$

for all $j \in \{ 1, \ldots , M - 1 \} $. Hence, we get

$$ \begin{aligned} & ( 1 + \beta _{j + 1} \cdot \Delta _{j + 1} ) \cdot ( \alpha _{j + 1} \cdot \Delta _{j + 1} ) \cdot I_{j + 1}^{2} + ( 1 + \beta _{j + 1} \cdot \Delta _{j + 1} ) \cdot N \cdot I_{j + 1} \\ &\quad = \alpha _{j + 1} \cdot \Delta _{j + 1} \cdot ( S_{j} + I_{j} ) \cdot I_{j + 1} + N \cdot I_{j}, \end{aligned} $$

and by setting

20 $$ A_{j + 1} := ( 1 + \beta _{j + 1} \cdot \Delta _{j + 1} ) \cdot ( \alpha _{j + 1} \cdot \Delta _{j + 1} ) $$

and

21 $$ B_{j + 1} := \frac{ ( 1 + \beta _{j + 1} \cdot \Delta _{j + 1} ) \cdot N - \alpha _{j + 1} \cdot \Delta _{j + 1} \cdot ( S_{j} + I_{j} )}{2}, $$

we get $A_{j + 1} \cdot I_{j + 1}^{2} + 2 \cdot B_{j + 1} \cdot I_{j + 1} = N \cdot I_{j}$ and can finally conclude

22 $$ I_{j + 1} = - \frac{B_{j + 1}}{A_{j + 1}} + \sqrt{ \frac{B_{j + 1}^{2}}{A_{j + 1}^{2}} + \frac{N \cdot I_{j}}{A_{j + 1}}} $$

for all $j \in \{ 1, \ldots , M - 1 \} $. We now have an explicit solution formula for $I_{j + 1}$ for all $j \in \{ 1, \ldots , M - 1 \} $ and therefore also for $S_{j + 1}$ and $R_{j + 1}$ for all $j \in \{ 1, \ldots , M - 1 \} $. Summarizing our results, we can formulate the following theorem.

#### Theorem 9

*Let us assume that* $0 < \alpha _{\mathrm{min}} \leq a_{j} \leq \alpha _{\mathrm{max}} < 1$ *and* $0 < \beta _{\mathrm{min}} \leq \beta _{j} \leq \beta _{\mathrm{max}} < 1$ *are given for all* $j \in \{ 1, \ldots , M \} $, *that* $0 < t_{j + 1} - t_{j} \leq 1$ *holds for all* $j \in \{ 1, \ldots , M - 1 \} $, *and that* $S_{1} > 0$, $I_{1} > 0$, *and* $R_{1} \geq 0$ *are prescribed*. *The implicit solution scheme* (17)

$$ \textstyle\begin{cases} S_{j + 1} = \frac{S_{j}}{1 + \alpha _{j + 1} \cdot ( t_{j + 1} - t_{j} ) \cdot \frac{I_{j + 1}}{N}}, \\ I_{j + 1} = \frac{I_{j}}{1 + \beta _{j + 1} \cdot ( t_{j + 1} - t_{j} ) - \alpha _{j + 1} \cdot ( t_{j + 1} - t_{j} ) \cdot \frac{S_{j + 1}}{N}}, \\ R_{j + 1} = R_{j} + \beta _{j + 1} \cdot ( t_{j + 1} - t_{j} ) \cdot I_{j + 1} \end{cases} $$

*is uniquely solvable for all* $j \in \{ 1, \ldots , M - 1 \} $. *It holds* (22)

$$ I_{j + 1} = - \frac{B_{j + 1}}{A_{j + 1}} + \sqrt{ \frac{B_{j + 1}^{2}}{A_{j + 1}^{2}} + \frac{N \cdot I_{j}}{A_{j + 1}}} $$

*for all* $j \in \{ 1, \ldots , M - 1 \} $ *with*

$$ A_{j + 1} := ( 1 + \beta _{j + 1} \cdot \Delta _{j + 1} ) \cdot ( \alpha _{j + 1} \cdot \Delta _{j + 1} ) $$

*and*

$$ B_{j + 1} := \frac{ ( 1 + \beta _{j + 1} \cdot \Delta _{j + 1} ) \cdot N - \alpha _{j + 1} \cdot \Delta _{j + 1} \cdot ( S_{j} + I_{j} )}{2}, $$

*from* (20) *and* (21).

### Monotonicity properties and long-time behavior

We show that many of the continuous properties from Theorems 4 and 8 even translate to the time-discrete implicit scheme (17).

#### Theorem 10

*For our time*-*discrete implicit solution scheme* (17), *we have*:

1. $0 \leq I_{j} \leq N$ *for all* $j \in \{ 1, \ldots , M \} $;
2. $0 \leq S_{j} \leq N$ *for all* $j \in \{ 1, \ldots , M \} $ *and* $S_{j + 1} \leq S_{j}$ *for all* $j \in \{ 1, \ldots , M - 1 \} $;
3. $0 \leq R_{j} \leq N$ *for all* $j \in \{ 1, \ldots , M \} $ *and* $R_{j + 1} \geq R_{j}$ *for all* $j \in \{ 1, \ldots , M - 1 \} $;
4. $\lim_{j \to \infty } I_{j} = 0$.

#### Proof

1) It holds $I_{j} \geq 0$ due to (22) and $I_{j} \leq N$ due to (16) for all $j \in \{ 1, \ldots , M \} $.

2) By our first property and due to (16), we have the inequality $0 \leq S_{j} \leq N$ for all $j \in \{ 1, \ldots , M \} $. Again by our first property, we obtain

$$ S_{j + 1} = \frac{S_{j}}{1 + \alpha _{j + 1} \cdot \Delta _{j + 1} \cdot \frac{I_{j + 1}}{N}} \leq S_{j} $$

for all $j \in \{ 1, \ldots , M - 1 \} $.

3) By our first property and due to (16), we obtain the inequality $0 \leq R_{j} \leq N$ for all $j \in \{ 1, \ldots , M \} $. Again by our first property, we conclude

$$ R_{j + 1} = R_{j} + \beta _{j + 1} \cdot \Delta _{j + 1} \cdot I_{j + 1} \geq R_{j} $$

for all $j \in \{ 1, \ldots , M - 1 \} $.

4) Since $\{ R_{j} \} _{j \in \mathbb{N}}$ is monotonically increasing and bounded above by the total population size *N*, there exists a nonnegative constant $R^{\star }$ such that $\lim_{j \to \infty } R_{j} = R^{\star }$. Furthermore, it holds

$$ R_{j + 1} - R_{j} = \beta _{j + 1} \cdot \Delta _{j + 1} \cdot I_{j + 1}, $$

which yields

$$ I_{j + 1} \leq \frac{R_{j + 1} - R_{j}}{\beta _{\mathrm{min}} \cdot \Delta _{j + 1}}. $$

This implies $\lim_{j \to \infty } I_{j} = 0$ and completes our assertion’s proof. □

### Error analysis

Now, we want to provide an upper bound for error propagation. Before proving this statement, we need to formulate some assumptions for our convergence analysis. We summarize them in the following list:

1. Let $[ 0, T ]$ be the considered time interval where $t_{1} = 0 < t_{2} < \cdots < t_{M - 1} < t_{M} = T$;
2. Let the initial conditions of the time-continuous and the time-discrete models coincide;
3. Let the solution functions *S*, *I*, $R \colon [ 0, T ] \longrightarrow [ 0, \infty )$ be twice continuously differentiable;
4. Let the time-varying transmission rate $\alpha \colon [ 0, T ] \longrightarrow [ 0, \infty )$ and the time-varying recovery rate $\beta \colon [ 0, T ] \longrightarrow [ 0, \infty )$ be once continuously differentiable;
5. Let the time-varying transmission and recovery rates be bounded, i.e., there are nonnegative constants $\alpha _{\mathrm{min}}$, $\alpha _{\mathrm{max}}$, $\beta _{\mathrm{min}}$, $\beta _{\mathrm{max}}$ such that $0 < \alpha _{\mathrm{min}} \leq \alpha ( t ) \leq \alpha _{\mathrm{max}} < 1$ and $0 < \beta _{\mathrm{min}} \leq \beta ( t ) \leq \beta _{ \mathrm{max}} < 1$ hold for all $t \in [ 0, T ]$;
6. Choose $\Delta _{p} < \min \{ \frac{1}{4 \cdot ( \alpha _{\mathrm{max}} + \beta _{\mathrm{max}} )}, 1 \} \leq 1$ for all $p \in \mathbb{N}$ and set $\Delta := \max_{p \in \mathbb{N}} \Delta _{p}$.

Under these conditions, we obtain the following theorem where we adapt ideas from the error analysis of an explicit-implicit solution algorithm as presented in [20].

#### Theorem 11

*If the aforementioned assumptions are fulfilled*, *the difference between the solution of the time*-*continuous problem formulation* (8) *and the solution of the time*-*discrete problem formulation* (17) *fulfills*

23 $$ \bigl\lVert \mathbf{z}_{p + 1} - \mathbf{z} ( t_{p + 1} ) \bigr\rVert _{\infty } \leq C_{\mathrm{loc}} \cdot \Delta \cdot \biggl\{ \biggl( \frac{1}{1 - 2 \cdot ( \alpha _{\mathrm{max}} + \beta _{\mathrm{max}} ) \cdot \Delta } \biggr)^{p} - 1 \biggr\} . $$

#### Proof

We briefly describe our strategy first because this proof is technical. We begin with an estimation of local errors between time-continuous and time-discrete solutions. Afterwards, we consider error propagation in time. Conclusively, we investigate the cumulation of these errors. Time-discrete solutions are written as $S_{p}$ at time $t_{p}$ and time-continuous solutions as $S ( t_{p} )$ at the same time.

1) For examination of local errors, we assume that

$$ ( t_{p}, S_{p} )^{T} = \bigl( t_{p}, S ( t_{p} ) \bigr)^{T} ,\qquad ( t_{p}, I_{p} )^{T} = \bigl( t_{p}, I ( t_{p} ) \bigr)^{T} ,\qquad ( t_{p}, R_{p} )^{T} = \bigl( t_{p}, R ( t_{p} ) \bigr)^{T} $$

hold for arbitrary $p \in \{ 1, \ldots , M - 1 \} $ on the time interval $[ t_{p}, t_{p + 1} ]$. Here, we consider solely one time step and denote corresponding time-discrete solutions by $\widetilde{S_{p + 1}}$, $\widetilde{I_{p + 1}}$, and $\widetilde{R_{p + 1}}$.

1.1) It first holds

$$ \widetilde{S_{p + 1}} = \frac{S_{p}}{1 + \alpha _{p + 1} \cdot \Delta _{p + 1} \cdot \frac{\widetilde{I_{p + 1}}}{N}} = S ( t_{p} ) - \frac{\alpha _{p + 1} \cdot \Delta _{p + 1} \cdot \frac{\widetilde{I_{p + 1}}}{N} \cdot S ( t_{p} )}{1 + \alpha _{p + 1} \cdot \Delta _{p + 1} \cdot \frac{\widetilde{I_{p + 1}}}{N}}, $$

and this implies

$$\begin{aligned}& \bigl\vert S ( t_{p + 1} ) - \widetilde{S_{p + 1}} \bigr\vert \\& \quad = \biggl\vert S ( t_{p + 1} ) - S ( t_{p} ) + \frac{\alpha _{p + 1} \cdot \Delta _{p + 1} \cdot \frac{\widetilde{I_{p + 1}}}{N} \cdot S ( t_{p} )}{1 + \alpha _{p + 1} \cdot \Delta _{p + 1} \cdot \frac{\widetilde{I_{p + 1}}}{N}} \biggr\vert \\& \quad = \biggl\vert \int _{t_{p}}^{t_{p + 1}} S^{\prime } ( \tau ) \, \mathrm{d}\tau + \frac{\alpha _{p + 1} \cdot \Delta _{p + 1} \cdot \frac{\widetilde{I_{p + 1}}}{N} \cdot S ( t_{p} )}{1 + \alpha _{p + 1} \cdot \Delta _{p + 1} \cdot \frac{\widetilde{I_{p + 1}}}{N}} \biggr\vert \\& \quad = \biggl\vert \int _{t_{p}}^{t_{p + 1}} S^{\prime } ( \tau ) \, \mathrm{d}\tau - \Delta _{p + 1} \cdot S^{\prime } ( t_{p} ) + \Delta _{p + 1} \cdot S^{\prime } ( t_{p} ) + \frac{\alpha _{p + 1} \cdot \Delta _{p + 1} \cdot \frac{\widetilde{I_{p + 1}}}{N} \cdot S ( t_{p} )}{1 + \alpha _{p + 1} \cdot \Delta _{p + 1} \cdot \frac{\widetilde{I_{p + 1}}}{N}} \biggr\vert \\& \quad \leq \underbrace{ \biggl\vert \int _{t_{p}}^{t_{p + 1}} S^{\prime } ( \tau ) \, \mathrm{d}\tau - \Delta _{p + 1} \cdot S^{\prime } ( t_{p} ) \biggr\vert }_{:= I_{\mathrm{S,1}}} + \underbrace{ \biggl\vert \Delta _{p + 1} \cdot S^{\prime } ( t_{p} ) + \frac{\alpha _{p + 1} \cdot \Delta _{p + 1} \cdot \frac{\widetilde{I_{p + 1}}}{N} \cdot S ( t_{p} )}{1 + \alpha _{p + 1} \cdot \Delta _{p + 1} \cdot \frac{\widetilde{I_{p + 1}}}{N}} \biggr\vert }_{:= \mathit{II}_{\mathrm{S,1}}} \end{aligned}$$

by application of the triangle inequality. We estimate these terms separately. For $I_{\mathrm{S,1}}$, we obtain

$$\begin{aligned} I_{\mathrm{S,1}} = & \biggl\vert \int _{t_{p}}^{t_{p + 1}} S^{ \prime } ( \tau ) \, \mathrm{d}\tau - \Delta _{p + 1} \cdot S^{\prime } ( t_{p} ) \biggr\vert = \biggl\vert \int _{t_{p}}^{t_{p + 1}} \bigl\{ S^{\prime } ( \tau ) - S^{\prime } ( t_{p} ) \bigr\} \, \mathrm{d}\tau \biggr\vert \\ = & \biggl\vert \int _{t_{p}}^{t_{p + 1}} ( \tau - t_{p} ) \cdot \frac{S^{\prime } ( \tau ) - S^{\prime } ( t_{p} )}{\tau - t_{p}} \, \mathrm{d}\tau \biggr\vert \end{aligned}$$

and the mean value theorem of calculus implies the existence of $\xi _{\mathrm{S,1}} \in ( t_{p}, t_{p + 1} )$ such that

$$ \bigl\vert S^{\prime \prime } ( \xi _{\mathrm{S,1}} ) \bigr\vert = \biggl\vert \frac{S^{\prime } ( \tau ) - S^{\prime } ( t_{p} )}{\tau - t_{p}} \biggr\vert \leq \bigl\lVert S^{\prime \prime } ( t ) \bigr\rVert _{ \infty } $$

holds. This yields

24 $$ I_{\mathrm{S,1}} \leq \bigl\lVert S^{\prime \prime } ( t ) \bigr\rVert _{\infty } \cdot \int _{t_{p}}^{t_{p + 1}} ( \tau - t_{p} ) \, \mathrm{d}\tau = \frac{1}{2} \cdot \Delta _{p + 1}^{2} \cdot \bigl\lVert S^{\prime \prime } ( t ) \bigr\rVert _{ \infty }. $$

For $\mathit{II}_{S,1}$, we see that

$$\begin{aligned} \mathit{II}_{\mathrm{S,1}} = & \biggl\vert \Delta _{p + 1} \cdot S^{\prime } ( t_{p} ) + \frac{\alpha _{p + 1} \cdot \Delta _{p + 1} \cdot \frac{\widetilde{I_{p + 1}}}{N} \cdot S ( t_{p} )}{1 + \alpha _{p + 1} \cdot \Delta _{p + 1} \cdot \frac{\widetilde{I_{p + 1}}}{N}} \biggr\vert \\ = & \biggl\vert - \alpha _{p} \cdot \Delta _{p + 1} \cdot \frac{S ( t_{p} ) \cdot I ( t_{p} )}{N} + \frac{\alpha _{p + 1} \cdot \Delta _{p + 1} \cdot \frac{\widetilde{I_{p + 1}}}{N} \cdot S ( t_{p} )}{1 + \alpha _{p + 1} \cdot \Delta _{p + 1} \cdot \frac{\widetilde{I_{p + 1}}}{N}} \biggr\vert \\ = & \biggl\vert \frac{\Delta _{p + 1} \cdot S ( t_{p} )}{N} \biggr\vert \cdot \biggl\vert - \alpha _{p} \cdot I ( t_{p} ) + \frac{\alpha _{p + 1} \cdot \widetilde{I_{p + 1}}}{1 + \alpha _{p + 1} \cdot \Delta _{p + 1} \cdot \frac{\widetilde{I_{p + 1}}}{N}} \biggr\vert \\ \leq & \Delta _{p + 1} \cdot \biggl\vert \frac{- \alpha _{p} \cdot I ( t_{p} ) \cdot \{ 1 + \alpha _{p + 1} \cdot \Delta _{p + 1} \cdot \frac{\widetilde{I_{p + 1}}}{N} \} + \alpha _{p + 1} \cdot \widetilde{I_{p + 1}}}{1 + \alpha _{p + 1} \cdot \Delta _{p + 1} \cdot \frac{\widetilde{I_{p + 1}}}{N}} \biggr\vert \\ \leq & \Delta _{p + 1} \cdot \biggl\vert - \alpha _{p} \cdot I ( t_{p} ) \cdot \biggl\{ 1 + \alpha _{p + 1} \cdot \Delta _{p + 1} \cdot \frac{\widetilde{I_{p + 1}}}{N} \biggr\} + \alpha _{p + 1} \cdot \widetilde{I_{p + 1}} \biggr\vert \\ = & \Delta _{p + 1} \cdot \biggl\vert \alpha _{p + 1} \cdot \widetilde{I_{p + 1}} - \alpha _{p} \cdot I ( t_{p} ) \cdot \biggl\{ 1 + \alpha _{p + 1} \cdot \Delta _{p + 1} \cdot \frac{\widetilde{I_{p + 1}}}{N} \biggr\} \biggr\vert \\ \leq & \Delta _{p + 1} \cdot \bigl\vert \alpha _{p + 1} \cdot \widetilde{I_{p + 1}} - \alpha _{p} \cdot I ( t_{p} ) \bigr\vert + \Delta _{p + 1} \cdot \biggl\vert \alpha _{p} \cdot I ( t_{p} ) \cdot \alpha _{p + 1} \cdot \Delta _{p + 1} \cdot \frac{\widetilde{I_{p + 1}}}{N} \biggr\vert \\ \leq & \underbrace{\Delta _{p + 1} \cdot \bigl\vert \alpha _{p + 1} \cdot \widetilde{I_{p + 1}} - \alpha _{p} \cdot I ( t_{p} ) \bigr\vert }_{:= \mathit{III}_{\mathrm{S,1}}} + \Delta _{p + 1}^{2} \cdot \alpha _{\mathrm{max}}^{2} \cdot N \end{aligned}$$

is valid by the definition of $S^{\prime } ( t )$, boundedness of our solution functions, and application of the triangle inequality. By plugging

$$ \widetilde{I_{p + 1}} = \frac{I ( t_{p} )}{1 + \beta _{p + 1} \cdot \Delta _{p + 1} - \alpha _{p + 1} \cdot \Delta _{p + 1} \cdot \frac{\widetilde{S_{p + 1}}}{N}} $$

into $\mathit{III}_{\mathrm{S,1}}$, we obtain

$$\begin{aligned} \mathit{III}_{\mathrm{S,1}} = & \Delta _{p + 1} \cdot \bigl\vert \alpha _{p + 1} \cdot \widetilde{I_{p + 1}} - \alpha _{p} \cdot I ( t_{p} ) \bigr\vert \\ = & \Delta _{p + 1} \cdot \biggl\vert \alpha _{p} \cdot I ( t_{p} ) - \alpha _{p + 1} \cdot \frac{I ( t_{p} )}{1 + \beta _{p + 1} \cdot \Delta _{p + 1} - \alpha _{p + 1} \cdot \Delta _{p + 1} \cdot \frac{\widetilde{S_{p + 1}}}{N}} \biggr\vert \\ = & \Delta _{p + 1} \cdot \bigl\vert I ( t_{p} ) \bigr\vert \cdot \biggl\vert \alpha _{p} - \frac{\alpha _{p + 1}}{1 + \beta _{p + 1} \cdot \Delta _{p + 1} - \alpha _{p + 1} \cdot \Delta _{p + 1} \cdot \frac{\widetilde{S_{p + 1}}}{N}} \biggr\vert \\ \leq & \Delta _{p + 1} \cdot N \cdot \biggl\vert \frac{\alpha _{p} \cdot \{ 1 + \beta _{p + 1} \cdot \Delta _{p + 1} - \alpha _{p + 1} \cdot \Delta _{p + 1} \cdot \frac{\widetilde{S_{p + 1}}}{N} \} - \alpha _{p + 1}}{1 + \beta _{p + 1} \cdot \Delta _{p + 1} - \alpha _{p + 1} \cdot \Delta _{p + 1} \cdot \frac{\widetilde{S_{p + 1}}}{N}} \biggr\vert \\ \leq & \frac{\Delta _{p + 1} \cdot N}{1 - \alpha _{\mathrm{max}}} \cdot \biggl\vert \alpha _{p} \cdot \biggl\{ 1 + \beta _{p + 1} \cdot \Delta _{p + 1} - \alpha _{p + 1} \cdot \Delta _{p + 1} \cdot \frac{\widetilde{S_{p + 1}}}{N} \biggr\} - \alpha _{p + 1} \biggr\vert \\ \leq & \frac{\Delta _{p + 1} \cdot N}{1 - \alpha _{\mathrm{max}}} \cdot \biggl\{ \vert \alpha _{p} - \alpha _{p + 1} \vert + \biggl\vert \alpha _{p} \cdot \biggl\{ \beta _{p + 1} \cdot \Delta _{p + 1} - \alpha _{p + 1} \cdot \Delta _{p + 1} \cdot \frac{\widetilde{S_{p + 1}}}{N} \biggr\} \biggr\vert \biggr\} \\ = & \frac{\Delta _{p + 1}^{2} \cdot N}{1 - \alpha _{\mathrm{max}}} \cdot \biggl\{ \biggl\vert \frac{\alpha _{p + 1} - \alpha _{p}}{t_{p + 1} - t_{p}} \biggr\vert + \biggl\vert \alpha _{p} \cdot \biggl\{ \beta _{p + 1} - \alpha _{p + 1} \cdot \frac{\widetilde{S_{p + 1}}}{N} \biggr\} \biggr\vert \biggr\} \end{aligned}$$

by the boundedness of our solution functions and application of the triangle inequality. By the mean value theorem, there exists $\xi _{\alpha ,\mathrm{1}} \in [ t_{p}, t_{p + 1} ]$ such that

$$ \bigl\vert \alpha ^{\prime } ( \xi _{\alpha ,\mathrm{1}} ) \bigr\vert = \biggl\vert \frac{\alpha _{p + 1} - \alpha _{p}}{t_{p + 1} - t_{p}} \biggr\vert \leq \bigl\lVert \alpha ^{\prime } ( t ) \bigr\rVert _{\infty } $$

holds. Hence, an additional application of the triangle inequality and boundedness of the solution functions yields

25 $$ \mathit{III}_{\mathrm{S,1}} \leq \frac{\Delta _{p + 1}^{2} \cdot N}{1 - \alpha _{\mathrm{max}}} \cdot \bigl\{ \bigl\lVert \alpha ^{\prime } ( t ) \bigr\rVert _{ \infty } + \alpha _{\mathrm{max}} \cdot ( \alpha _{\mathrm{max}} + \beta _{\mathrm{max}} ) \bigr\} . $$

Plugging (25) into $\mathit{II}_{\mathrm{S,1}}$, we conclude that

26 $$ \mathit{II}_{\mathrm{S,1}} \leq \frac{\Delta _{p + 1}^{2} \cdot N}{1 - \alpha _{\mathrm{max}}} \cdot \bigl\{ \bigl\lVert \alpha ^{\prime } ( t ) \bigr\rVert _{ \infty } + \alpha _{\mathrm{max}} \cdot ( \alpha _{\mathrm{max}} + \beta _{\mathrm{max}} ) \bigr\} + \Delta _{p + 1}^{2} \cdot \alpha _{\mathrm{max}}^{2} \cdot N $$

holds. Combining (24) and (26), we infer that

$$\begin{aligned}& \bigl\vert S ( t_{p + 1} ) - \widetilde{S_{p + 1}} \bigr\vert \\& \quad \leq I_{\mathrm{S,1}} + \mathit{II}_{\mathrm{S,1}} \\& \quad \leq \frac{1}{2} \cdot \Delta _{p + 1}^{2} \cdot \bigl\lVert S^{ \prime \prime } ( t ) \bigr\rVert _{\infty } + \frac{\Delta _{p + 1}^{2} \cdot N}{1 - \alpha _{\mathrm{max}}} \cdot \bigl\{ \bigl\lVert \alpha ^{\prime } ( t ) \bigr\rVert _{ \infty } + \alpha _{\mathrm{max}} \cdot ( \alpha _{\mathrm{max}} + \beta _{\mathrm{max}} ) \bigr\} \\& \qquad {}+ \Delta _{p + 1}^{2} \cdot \alpha _{\mathrm{max}}^{2} \cdot N \\& \quad = \Delta _{p + 1}^{2} \cdot \underbrace{ \biggl\{ \frac{1}{2} \cdot \bigl\lVert S^{\prime \prime } ( t ) \bigr\rVert _{\infty } + \frac{N \cdot \{ \lVert \alpha ^{\prime } ( t ) \rVert _{\infty } + \alpha _{\mathrm{max}} \cdot ( \alpha _{\mathrm{max}} + \beta _{\mathrm{max}} ) \} }{1 - \alpha _{\mathrm{max}}} + N \cdot \alpha _{\mathrm{max}}^{2} \biggr\} }_{=: C_{\mathrm{loc,S}}} \end{aligned}$$

is valid. Thus, it holds

27 $$ \bigl\vert S ( t_{p + 1} ) - \widetilde{S_{p + 1}} \bigr\vert \leq C_{\mathrm{loc,S}} \cdot \Delta _{p + 1}^{2}. $$

1.2) We observe that

$$\begin{aligned} \widetilde{I_{p + 1}} = & \frac{I_{p}}{1 + \beta _{p + 1} \cdot \Delta _{p + 1} - \alpha _{p + 1} \cdot \Delta _{p + 1} \cdot \frac{\widetilde{S_{p + 1}}}{N}} \\ = & \frac{I ( t_{p} )}{1 + \beta _{p + 1} \cdot \Delta _{p + 1} - \alpha _{p + 1} \cdot \Delta _{p + 1} \cdot \frac{\widetilde{S_{p + 1}}}{N}} \\ = & I ( t_{p} ) - I ( t_{p} ) \cdot \frac{\beta _{p + 1} \cdot \Delta _{p + 1} - \alpha _{p + 1} \cdot \Delta _{p + 1} \cdot \frac{\widetilde{S_{p + 1}}}{N}}{1 + \beta _{p + 1} \cdot \Delta _{p + 1} - \alpha _{p + 1} \cdot \Delta _{p + 1} \cdot \frac{\widetilde{S_{p + 1}}}{N}} \end{aligned}$$

is valid. Hence, it follows

$$\begin{aligned}& \bigl\vert I ( t_{p + 1} ) - \widetilde{I_{p + 1}} \bigr\vert \\& \quad = \biggl\vert I ( t_{p + 1} ) - I ( t_{p} ) + I ( t_{p} ) \cdot \frac{\beta _{p + 1} \cdot \Delta _{p + 1} - \alpha _{p + 1} \cdot \Delta _{p + 1} \cdot \frac{\widetilde{S_{p + 1}}}{N}}{1 + \beta _{p + 1} \cdot \Delta _{p + 1} - \alpha _{p + 1} \cdot \Delta _{p + 1} \cdot \frac{\widetilde{S_{p + 1}}}{N}} \biggr\vert \\& \quad = \biggl\vert \int _{t_{p}}^{t_{p + 1}} I^{\prime } ( \tau ) \, \mathrm{d}\tau + I ( t_{p} ) \cdot \frac{\beta _{p + 1} \cdot \Delta _{p + 1} - \alpha _{p + 1} \cdot \Delta _{p + 1} \cdot \frac{\widetilde{S_{p + 1}}}{N}}{1 + \beta _{p + 1} \cdot \Delta _{p + 1} - \alpha _{p + 1} \cdot \Delta _{p + 1} \cdot \frac{\widetilde{S_{p + 1}}}{N}} \biggr\vert \\& \quad \leq \biggl\vert \int _{t_{p}}^{t_{p + 1}} I^{\prime } ( \tau ) \, \mathrm{d}\tau - \Delta _{p + 1} \cdot I^{\prime } ( t_{p} ) \biggr\vert \\& \qquad {}+ \biggl\vert \Delta _{p + 1} \cdot I^{\prime } ( t_{p} ) + I ( t_{p} ) \cdot \frac{\beta _{p + 1} \cdot \Delta _{p + 1} - \alpha _{p + 1} \cdot \Delta _{p + 1} \cdot \frac{\widetilde{S_{p + 1}}}{N}}{1 + \beta _{p + 1} \cdot \Delta _{p + 1} - \alpha _{p + 1} \cdot \Delta _{p + 1} \cdot \frac{\widetilde{S_{p + 1}}}{N}} \biggr\vert \\& \quad \leq \frac{\Delta _{p + 1}^{2}}{2} \cdot \bigl\lVert I^{\prime \prime } ( t ) \bigr\rVert _{\infty } + \underbrace{ \biggl\vert \Delta _{p + 1} \cdot I^{\prime } ( t_{p} ) + I ( t_{p} ) \cdot \frac{\beta _{p + 1} \cdot \Delta _{p + 1} - \alpha _{p + 1} \cdot \Delta _{p + 1} \cdot \frac{\widetilde{S_{p + 1}}}{N}}{1 + \beta _{p + 1} \cdot \Delta _{p + 1} - \alpha _{p + 1} \cdot \Delta _{p + 1} \cdot \frac{\widetilde{S_{p + 1}}}{N}} \biggr\vert }_{=: I_{\mathrm{I,1}}} \end{aligned}$$

by application of the triangle inequality and with similar arguments as provided in the previous step. As

$$ I^{\prime } ( t_{p} ) = \frac{\alpha _{p} \cdot I ( t_{p} ) \cdot S ( t_{p} )}{N} - \beta _{p} \cdot I ( t_{p} ) $$

holds, we further obtain

$$\begin{aligned} I_{\mathrm{I,1}} = & \biggl\vert \Delta _{p + 1} \cdot I^{\prime } ( t_{p} ) + I ( t_{p} ) \cdot \frac{\beta _{p + 1} \cdot \Delta _{p + 1} - \alpha _{p + 1} \cdot \Delta _{p + 1} \cdot \frac{\widetilde{S_{p + 1}}}{N}}{1 + \beta _{p + 1} \cdot \Delta _{p + 1} - \alpha _{p + 1} \cdot \Delta _{p + 1} \cdot \frac{\widetilde{S_{p + 1}}}{N}} \biggr\vert \\ = & \Delta _{p + 1} \cdot \biggl\vert I^{\prime } ( t_{p} ) + \frac{\beta _{p + 1} \cdot I ( t_{p} ) - \alpha _{p + 1} \cdot I ( t_{p} ) \cdot \frac{\widetilde{S_{p + 1}}}{N}}{1 + \beta _{p + 1} \cdot \Delta _{p + 1} - \alpha _{p + 1} \cdot \Delta _{p + 1} \cdot \frac{\widetilde{S_{p + 1}}}{N}} \biggr\vert \\ = & \Delta _{p + 1} \cdot \biggl\vert \frac{\alpha _{p} \cdot I ( t_{p} ) \cdot S ( t_{p} )}{N} - \beta _{p} \cdot I ( t_{p} ) + \frac{\beta _{p + 1} \cdot I ( t_{p} ) - \alpha _{p + 1} \cdot I ( t_{p} ) \cdot \frac{\widetilde{S_{p + 1}}}{N}}{1 + \beta _{p + 1} \cdot \Delta _{p + 1} - \alpha _{p + 1} \cdot \Delta _{p + 1} \cdot \frac{\widetilde{S_{p + 1}}}{N}} \biggr\vert \\ \leq & \frac{\Delta _{p + 1}}{1 - \alpha _{\mathrm{max}}} \cdot \biggl\vert \alpha _{p} \cdot \frac{I ( t_{p} ) \cdot S ( t_{p} )}{N} - \alpha _{p + 1} \cdot \frac{I ( t_{p} ) \cdot \widetilde{S_{p + 1}}}{N} + \beta _{p + 1} \cdot I ( t_{p} ) - \beta _{p} \cdot I ( t_{p} ) \biggr\vert \\ & {}+ \frac{\Delta _{p + 1}^{2} \cdot N}{1 - \alpha _{\mathrm{max}}} \cdot \biggl\vert \biggl( \beta _{p + 1} - \alpha _{p + 1} \cdot \frac{\widetilde{S_{p + 1}}}{N} \biggr) \cdot \biggl( \alpha _{p} \cdot \frac{S ( t_{p} )}{N} - \beta _{p} \biggr) \biggr\vert \\ \leq & \frac{\Delta _{p + 1}}{1 - \alpha _{\mathrm{max}}} \cdot \bigl\vert \alpha _{p} \cdot S ( t_{p} ) - \alpha _{p + 1} \cdot \widetilde{S_{p + 1}} \bigr\vert + \frac{\Delta _{p + 1}}{1 - \alpha _{\mathrm{max}}} \cdot \bigl\vert \beta _{p + 1} \cdot I ( t_{p} ) - \beta _{p} \cdot I ( t_{p} ) \bigr\vert \\ & {}+ \frac{\Delta _{p + 1}^{2}}{1 - \alpha _{\mathrm{max}}} \cdot ( \alpha _{\mathrm{max}} + \beta _{\mathrm{max}} )^{2} \\ \leq & \frac{\Delta _{p + 1}}{1 - \alpha _{\mathrm{max}}} \cdot \underbrace{ \bigl\vert \alpha _{p} \cdot S ( t_{p} ) - \alpha _{p + 1} \cdot \widetilde{S_{p + 1}} \bigr\vert }_{=: \mathit{II}_{\mathrm{I,1}}} + \frac{\Delta _{p + 1} \cdot N}{1 - \alpha _{\mathrm{max}}} \cdot \underbrace{ \vert \beta _{p + 1} - \beta _{p} \vert }_{=: \mathit{III}_{ \mathrm{I,1}}} \\ & {}+ \frac{\Delta _{p + 1}^{2}}{1 - \alpha _{\mathrm{max}}} \cdot ( \alpha _{\mathrm{max}} + \beta _{\mathrm{max}} )^{2} \end{aligned}$$

by the boundedness of the solution functions and application of the triangle inequality. By using

$$ \widetilde{S_{p + 1}} = \frac{S ( t_{p} )}{1 + \alpha _{p + 1} \cdot \Delta _{p + 1} \cdot \frac{\widetilde{I_{p + 1}}}{N}}, $$

we obtain

28 $$ \begin{aligned} \mathit{II}_{\mathrm{I,1}} & = \bigl\vert \alpha _{p} \cdot S ( t_{p} ) - \alpha _{p + 1} \cdot \widetilde{S_{p + 1}} \bigr\vert \\ & = \biggl\vert \alpha _{p} \cdot S ( t_{p} ) - \alpha _{p + 1} \cdot \frac{S ( t_{p} )}{1 + \alpha _{p + 1} \cdot \Delta _{p + 1} \cdot \frac{\widetilde{I_{p + 1}}}{N}} \biggr\vert \\ & \leq \bigl\vert \alpha _{p} \cdot S ( t_{p} ) - \alpha _{p + 1} \cdot S ( t_{p} ) \bigr\vert + \Delta _{p + 1} \cdot \bigl\vert \alpha _{p} \cdot \alpha _{p + 1} \cdot S ( t_{p} ) \bigr\vert \\ & \leq N \cdot \bigl\lVert \alpha ^{\prime } ( t ) \bigr\rVert _{ \infty } \cdot \Delta _{p + 1} + N \cdot \alpha _{\mathrm{max}}^{2} \cdot \Delta _{p + 1} \end{aligned} $$

by the boundedness of the solution functions, the mean value theorem of calculus, and application of the triangle inequality. Additionally, it holds

29 $$ \begin{aligned} \mathit{III}_{\mathrm{I,1}} & = \vert \beta _{p + 1} - \beta _{p} \vert \\ & \leq \Delta _{p + 1} \cdot \bigl\lVert \beta ^{\prime } ( t ) \bigr\rVert _{\infty } \end{aligned} $$

by application of the mean value theorem of calculus. Combining (28) and (29) and plugging these results into

$$\begin{aligned} I_{\mathrm{I,1}} \leq & \frac{\Delta _{p + 1}}{1 - \alpha _{\mathrm{max}}} \cdot \mathit{II}_{ \mathrm{I,1}} + \frac{\Delta _{p + 1} \cdot N}{1 - \alpha _{\mathrm{max}}} \cdot \mathit{III}_{ \mathrm{I,1}} + \frac{\Delta _{p + 1}^{2}}{1 - \alpha _{\mathrm{max}}} \cdot ( \alpha _{\mathrm{max}} + \beta _{\mathrm{max}} )^{2} \end{aligned}$$

yields

$$\begin{aligned} I_{\mathrm{I,1}} \leq& \frac{\Delta _{p + 1}}{1 - \alpha _{\mathrm{max}}} \cdot \bigl\{ N \cdot \bigl\lVert \alpha ^{\prime } ( t ) \bigr\rVert _{ \infty } \cdot \Delta _{p + 1} + N \cdot \alpha _{\mathrm{max}}^{2} \cdot \Delta _{p + 1} \bigr\} \\ & {}+ \frac{\Delta _{p + 1} \cdot N}{1 - \alpha _{\mathrm{max}}} \cdot \Delta _{p + 1} \cdot \bigl\lVert \beta ^{\prime } ( t ) \bigr\rVert _{\infty } + \frac{\Delta _{p + 1}^{2}}{1 - \alpha _{\mathrm{max}}} \cdot ( \alpha _{\mathrm{max}} + \beta _{\mathrm{max}} )^{2} \\ =& \frac{\Delta _{p + 1}^{2}}{1 - \alpha _{\mathrm{max}}} \cdot \underbrace{ \bigl\{ N \cdot \bigl\lVert \alpha ^{\prime } ( t ) \bigr\rVert _{\infty } + N \cdot \alpha _{\mathrm{max}}^{2} + N \cdot \bigl\lVert \beta ^{\prime } ( t ) \bigr\rVert _{\infty } + ( \alpha _{\mathrm{max}} + \beta _{\mathrm{max}} )^{2} \bigr\} }_{=: C_{\mathrm{I,help,1}}}. \end{aligned}$$

Plugging this inequality into

$$ \bigl\vert I ( t_{p + 1} ) - \widetilde{I_{p + 1}} \bigr\vert \leq \frac{\Delta _{p + 1}^{2}}{2} \cdot \bigl\lVert I^{\prime \prime } ( t ) \bigr\rVert _{\infty } + I_{\mathrm{I,1}} $$

implies

30 $$ \bigl\vert I ( t_{p + 1} ) - \widetilde{I_{p + 1}} \bigr\vert \leq \Delta _{p + 1}^{2} \cdot \underbrace{ \biggl\{ \frac{1}{2} \cdot \bigl\lVert I^{\prime \prime } ( t ) \bigr\rVert _{\infty } + \frac{C_{\mathrm{I,help,1}}}{1 - \alpha _{\mathrm{max}}} \biggr\} }_{=: C_{\mathrm{loc,I}}}. $$

1.3) We see that

$$ \widetilde{R_{p + 1}} = R ( t_{p} ) + \beta _{p + 1} \cdot \Delta _{p + 1} \cdot \widetilde{I_{p + 1}} $$

holds. This implies

$$\begin{aligned}& \bigl\vert R ( t_{p + 1} ) - \widetilde{R_{p + 1}} \bigr\vert \\& \quad = \bigl\vert R ( t_{p + 1} ) - R ( t_{p} ) - \beta _{p + 1} \cdot \Delta _{p + 1} \cdot \widetilde{I_{p + 1}} \bigr\vert \\& \quad = \biggl\vert \int _{t_{p}}^{t_{p + 1}} R^{\prime } ( \tau ) \, \mathrm{d}\tau - \Delta _{p + 1} \cdot R^{\prime } ( t_{p} ) + \Delta _{p + 1} \cdot R^{\prime } ( t_{p} ) - \beta _{p + 1} \cdot \Delta _{p + 1} \cdot \widetilde{I_{p + 1}} \biggr\vert \\& \quad \leq \biggl\vert \int _{t_{p}}^{t_{p + 1}} \bigl( R^{\prime } ( \tau ) - R^{\prime } ( t_{p} ) \bigr) \, \mathrm{d}\tau \biggr\vert + \bigl\vert \Delta _{p + 1} \cdot R^{\prime } ( t_{p} ) - \beta _{p + 1} \cdot \Delta _{p + 1} \cdot \widetilde{I_{p + 1}} \bigr\vert \\& \quad \leq \frac{\Delta _{p + 1}^{2}}{2} \cdot \bigl\lVert R^{\prime \prime } ( t ) \bigr\rVert _{\infty } + \Delta _{p + 1} \cdot \bigl\vert R^{ \prime } ( t_{p} ) - \beta _{p + 1} \cdot \widetilde{I_{p + 1}} \bigr\vert . \end{aligned}$$

By applying

$$ R^{\prime } ( t_{p} ) = \beta _{p} \cdot I ( t_{p} ) $$

and

$$\begin{aligned} \widetilde{I_{p + 1}} = & \frac{I ( t_{p} )}{1 + \beta _{p + 1} \cdot \Delta _{p + 1} - \alpha _{p + 1} \cdot \Delta _{p + 1} \cdot \frac{\widetilde{S_{p + 1}}}{N}} \\ = & I ( t_{p} ) - \frac{I ( t_{p} ) \cdot \{ \beta _{p + 1} \cdot \Delta _{p + 1} - \alpha _{p + 1} \cdot \Delta _{p + 1} \cdot \frac{\widetilde{S_{p + 1}}}{N} \} }{1 + \beta _{p + 1} \cdot \Delta _{p + 1} - \alpha _{p + 1} \cdot \Delta _{p + 1} \cdot \frac{\widetilde{S_{p + 1}}}{N}}, \end{aligned}$$

we obtain

$$\begin{aligned}& \bigl\vert R^{\prime } ( t_{p} ) - \beta _{p + 1} \cdot \widetilde{I_{p + 1}} \bigr\vert \\& \quad = \biggl\vert \beta _{p} \cdot I ( t_{p} ) - \beta _{p + 1} \cdot I ( t_{p} ) + \beta _{p + 1} \cdot \frac{I ( t_{p} ) \cdot \{ \beta _{p + 1} \cdot \Delta _{p + 1} - \alpha _{p + 1} \cdot \Delta _{p + 1} \cdot \frac{\widetilde{S_{p + 1}}}{N} \} }{1 + \beta _{p + 1} \cdot \Delta _{p + 1} - \alpha _{p + 1} \cdot \Delta _{p + 1} \cdot \frac{\widetilde{S_{p + 1}}}{N}} \biggr\vert \\& \quad \leq N \cdot \vert \beta _{p} - \beta _{p + 1} \vert + \Delta _{p + 1} \cdot \frac{N \cdot \beta _{\mathrm{max}}^{2} + N \cdot \alpha _{\mathrm{max}} \cdot \beta _{\mathrm{max}}}{1 - \alpha _{\mathrm{max}}} \\& \quad \leq N \cdot \Delta _{p + 1} \cdot \bigl\lVert \beta ^{\prime } ( t ) \bigr\rVert _{\infty } + \Delta _{p + 1} \cdot \frac{N \cdot \beta _{\mathrm{max}}^{2} + N \cdot \alpha _{\mathrm{max}} \cdot \beta _{\mathrm{max}}}{1 - \alpha _{\mathrm{max}}} \\& \quad = \Delta _{p + 1} \cdot \underbrace{ \biggl\{ N \cdot \bigl\lVert \beta ^{\prime } ( t ) \bigr\rVert _{\infty } + \frac{N \cdot \beta _{\mathrm{max}}^{2} + N \cdot \alpha _{\mathrm{max}} \cdot \beta _{\mathrm{max}}}{1 - \alpha _{\mathrm{max}}} \biggr\} }_{=:C_{ \mathrm{R,help,1}}}. \end{aligned}$$

Plugging this inequality into

$$ \bigl\vert R ( t_{p + 1} ) - \widetilde{R_{p + 1}} \bigr\vert \leq \frac{\Delta _{p + 1}^{2}}{2} \cdot \bigl\lVert R^{\prime \prime } ( t ) \bigr\rVert _{\infty } + \Delta _{p + 1} \cdot \bigl\vert R^{ \prime } ( t_{p} ) - \beta _{p + 1} \cdot \widetilde{I_{p + 1}} \bigr\vert $$

yields

31 $$ \begin{aligned} \bigl\vert R ( t_{p + 1} ) - \widetilde{R_{p + 1}} \bigr\vert & \leq \frac{\Delta _{p + 1}^{2}}{2} \cdot \bigl\lVert R^{\prime \prime } ( t ) \bigr\rVert _{\infty } + \Delta _{p + 1} \cdot \bigl\vert R^{ \prime } ( t_{p} ) - \beta _{p + 1} \cdot \widetilde{I_{p + 1}} \bigr\vert \\ & \leq \frac{\Delta _{p + 1}^{2}}{2} \cdot \bigl\lVert R^{\prime \prime } ( t ) \bigr\rVert _{\infty } + \Delta _{p + 1}^{2} \cdot C_{ \mathrm{R,help,1}} \\ & = \Delta _{p + 1}^{2} \cdot \underbrace{ \biggl\{ \frac{1}{2} \cdot \bigl\lVert R^{\prime \prime } ( t ) \bigr\rVert _{\infty } + C_{\mathrm{R,help,1}} \biggr\} }_{=: C_{\mathrm{loc,R}}}. \end{aligned} $$

1.4) Define $C_{\mathrm{loc}} := \max \{ C_{\mathrm{loc,S}}; C_{ \mathrm{loc,I}}; C_{\mathrm{loc,R}} \} $. It holds

32 $$ \bigl\lVert \mathbf{z} ( t_{p + 1} ) - \widetilde{ \mathbf{z}_{p + 1}} \bigr\rVert _{\infty } \leq C_{\mathrm{loc}} \cdot \Delta _{p + 1}^{2} $$

for local errors on time intervals $[ t_{p}, t_{p + 1} ]$.

2) In reality, $( t_{p}, S_{p} )^{T}$, $( t_{p}, I_{p} )^{T}$, and $( t_{p}, R_{p} )^{T}$ do not exactly lie on the graph of the time-continuous solution. Therefore, we must examine how procedural errors such as $S_{p} - S ( t_{p} )$, $I_{p} - I ( t_{p} )$ or $R_{p} - R ( t_{p} )$ propagate to the $( p + 1 )$th time step. These investigations are carried out in step 2) and in step 3). By (9), we see that

$$ \mathbf{z}_{p + 1} - \mathbf{z} ( t_{p + 1} ) = \bigl( \mathbf{z}_{p} - \mathbf{z} ( t_{p} ) \bigr) + \Delta _{p + 1} \cdot \bigl\{ \mathbf{G} ( t_{p + 1}, \mathbf{z}_{p + 1} ) - \mathbf{G} \bigl( t_{p + 1}, \mathbf{z} ( t_{p + 1} ) \bigr) \bigr\} $$

holds, and this implies

$$\begin{aligned}& \bigl\lVert \mathbf{z}_{p + 1} - \mathbf{z} ( t_{p + 1} ) \bigr\rVert _{\infty } \\& \quad \leq \bigl\lVert \mathbf{z}_{p} - \mathbf{z} ( t_{p} ) \bigr\rVert _{\infty } + \Delta _{p + 1} \cdot \bigl\lVert \mathbf{G} ( t_{p + 1}, \mathbf{z}_{p + 1} ) - \mathbf{G} \bigl( t_{p + 1}, \mathbf{z} ( t_{p + 1} ) \bigr) \bigr\rVert _{\infty }. \end{aligned}$$

We see that

$$\begin{aligned}& \bigl\lVert \mathbf{G} ( t_{p + 1}, \mathbf{z}_{p + 1} ) - \mathbf{G} \bigl( t_{p + 1}, \mathbf{z} ( t_{p + 1} ) \bigr) \bigr\rVert _{\infty } \\& \quad = \left \Vert \begin{pmatrix} \frac{\alpha _{p + 1}}{N} \cdot \{ I ( t_{p + 1} ) \cdot S ( t_{p + 1} ) - I_{p + 1} \cdot S_{p + 1} \} \\ \frac{\alpha _{p + 1}}{N} \cdot \{ I_{p + 1} \cdot S_{p + 1} - I ( t_{p + 1} ) \cdot S ( t_{p + 1} ) \} + \beta _{p + 1} \cdot \{ I ( t_{p + 1} ) - I_{p + 1} \} \\ \beta _{p + 1} \cdot \{ I_{p + 1} - I ( t_{p + 1} ) \} \end{pmatrix} \right \Vert _{\infty } \\& \quad \leq \left \Vert \begin{pmatrix} \alpha _{p + 1} \cdot \{ \lVert I_{p + 1} - I ( t_{p + 1} ) \rVert _{\infty } + \lVert S_{p + 1} - S ( t_{p + 1} ) \rVert _{\infty } \} \\ \alpha _{p + 1} \cdot \{ \lVert I_{p + 1} - I ( t_{p + 1} ) \rVert _{\infty } + \lVert S_{p + 1} - S ( t_{p + 1} ) \rVert _{\infty } \} + \beta _{p + 1} \cdot \lVert I_{p + 1} - I ( t_{p + 1} ) \rVert _{\infty } \\ \beta _{p + 1} \cdot \lVert I_{p + 1} - I ( t_{p + 1} ) \rVert _{\infty } \end{pmatrix} \right \Vert _{\infty } \\& \quad \leq 2 \cdot ( \alpha _{\mathrm{max}} + \beta _{\mathrm{max}} ) \cdot \bigl\lVert \mathbf{z}_{p + 1} - \mathbf{z} ( t_{p + 1} ) \bigr\rVert _{\infty } \end{aligned}$$

holds, and this implies

$$ \bigl\lVert \mathbf{z}_{p + 1} - \mathbf{z} ( t_{p + 1} ) \bigr\rVert _{\infty } \leq \bigl\lVert \mathbf{z}_{p} - \mathbf{z} ( t_{p} ) \bigr\rVert _{\infty } + 2 \cdot ( \alpha _{\mathrm{max}} + \beta _{\mathrm{max}} ) \cdot \Delta _{p + 1} \cdot \bigl\lVert \mathbf{z}_{p + 1} - \mathbf{z} ( t_{p + 1} ) \bigr\rVert _{ \infty }. $$

Hence, we conclude

33 $$ \begin{aligned} \bigl\lVert \mathbf{z}_{p + 1} - \mathbf{z} ( t_{p + 1} ) \bigr\rVert _{\infty } & \leq \frac{1}{1 - 2 \cdot ( \alpha _{\mathrm{max}} + \beta _{\mathrm{max}} ) \cdot \Delta _{p + 1}} \cdot \bigl\lVert \mathbf{z}_{p} - \mathbf{z} ( t_{p} ) \bigr\rVert _{\infty } \\ & \leq \frac{1}{1 - 2 \cdot ( \alpha _{\mathrm{max}} + \beta _{\mathrm{max}} ) \cdot \Delta } \cdot \bigl\lVert \mathbf{z}_{p} - \mathbf{z} ( t_{p} ) \bigr\rVert _{\infty } \end{aligned} $$

with $\Delta := \max_{p \in \{ 1, \ldots , M - 1 \} } \Delta _{p + 1} < \frac{1}{4 \cdot ( \alpha _{\mathrm{max}} + \beta _{\mathrm{max}} )}$.

3) Now, we want to prove the upper error bound between the time-discrete solution and the time-continuous solution. At first, we notice that

$$\begin{aligned} \bigl\lVert \mathbf{z}_{2} - \mathbf{z} ( t_{2} ) \bigr\rVert _{ \infty } \leq & \lVert \mathbf{z}_{2} - \widetilde{\mathbf{z}_{2}} \rVert _{\infty } + \bigl\lVert \widetilde{\mathbf{z}_{2}} - \mathbf{z} ( t_{2} ) \bigr\rVert _{\infty } \\ \leq & \biggl( \frac{1}{1 - 2 \cdot ( \alpha _{\mathrm{max}} + \beta _{\mathrm{max}} ) \cdot \Delta } \biggr) \cdot \underbrace{\bigl\lVert \mathbf{z}_{1} - \mathbf{z} ( t_{1} ) \bigr\rVert _{\infty }}_{= 0} + C_{\mathrm{loc}} \cdot \Delta ^{2} \\ = & C_{\mathrm{loc}} \cdot \Delta ^{2} \end{aligned}$$

is valid for $p = 1$ by (32), by (33), and by our assumption that initial conditions of the time-continuous and the time-discrete models coincide. For $p = 2$, we obtain

$$\begin{aligned} \bigl\lVert \mathbf{z}_{3} - \mathbf{z} ( t_{3} ) \bigr\rVert _{ \infty } \leq & \lVert \mathbf{z}_{3} - \widetilde{ \mathbf{z}_{3}} \rVert _{\infty } + \bigl\lVert \widetilde{ \mathbf{z}_{3}} - \mathbf{z} ( t_{3} ) \bigr\rVert _{\infty } \\ \leq & \biggl( \frac{1}{1 - 2 \cdot ( \alpha _{\mathrm{max}} + \beta _{\mathrm{max}} ) \cdot \Delta } \biggr) \cdot \bigl\lVert \mathbf{z}_{2} - \mathbf{z} ( t_{2} ) \bigr\rVert _{\infty } + C_{\mathrm{loc}} \cdot \Delta ^{2} \\ \leq & \biggl( \frac{1}{1 - 2 \cdot ( \alpha _{\mathrm{max}} + \beta _{\mathrm{max}} ) \cdot \Delta } \biggr) \cdot \bigl\{ C_{\mathrm{loc}} \cdot \Delta ^{2} \bigr\} + C_{ \mathrm{loc}} \cdot \Delta ^{2} \\ = & C_{\mathrm{loc}} \cdot \Delta ^{2} \cdot \Biggl\{ \sum _{j = 0}^{3 - 2} \biggl( \frac{1}{1 - 2 \cdot ( \alpha _{\mathrm{max}} + \beta _{\mathrm{max}} ) \cdot \Delta } \biggr)^{j} \Biggr\} . \end{aligned}$$

For arbitrary $p \in \{ 1, \ldots , M - 2 \} $, we assume that

$$ \bigl\lVert \mathbf{z}_{p + 1} - \mathbf{z} ( t_{p + 1} ) \bigr\rVert _{\infty } \leq C_{\mathrm{loc}} \cdot \Delta ^{2} \cdot \Biggl\{ \sum_{j = 0}^{p - 1} \biggl( \frac{1}{1 - 2 \cdot ( \alpha _{\mathrm{max}} + \beta _{\mathrm{max}} ) \cdot \Delta } \biggr)^{j} \Biggr\} $$

is valid. This yields

$$\begin{aligned}& \bigl\lVert \mathbf{z}_{p + 2} - \mathbf{z} ( t_{p + 2} ) \bigr\rVert _{\infty } \\& \quad \leq \lVert \mathbf{z}_{p + 2} - \widetilde{\mathbf{z}_{p + 2}} \rVert _{\infty } + \bigl\lVert \widetilde{\mathbf{z}_{p + 2}} - \mathbf{z} ( t_{p + 2} ) \bigr\rVert _{\infty } \\& \quad \leq C_{\mathrm{loc}} \cdot \Delta ^{2} + \biggl( \frac{1}{1 - 2 \cdot ( \alpha _{\mathrm{max}} + \beta _{\mathrm{max}} ) \cdot \Delta } \biggr) \cdot \bigl\lVert \mathbf{z}_{p + 1} - \mathbf{z} ( t_{p + 1} ) \bigr\rVert _{\infty } \\& \quad \leq C_{\mathrm{loc}} \cdot \Delta ^{2} \cdot \Biggl\{ 1 + \sum_{j = 0}^{p - 1} \biggl( \frac{1}{1 - 2 \cdot ( \alpha _{\mathrm{max}} + \beta _{\mathrm{max}} ) \cdot \Delta } \biggr)^{j + 1} \Biggr\} \\& \quad = C_{\mathrm{loc}} \cdot \Delta ^{2} \cdot \Biggl\{ \sum _{j = 0}^{p} \biggl( \frac{1}{1 - 2 \cdot ( \alpha _{\mathrm{max}} + \beta _{\mathrm{max}} ) \cdot \Delta } \biggr)^{j} \Biggr\} \end{aligned}$$

by induction. By applying the geometric series, we obtain

$$\begin{aligned}& \bigl\lVert \mathbf{z}_{p + 1} - \mathbf{z} ( t_{p + 1} ) \bigr\rVert _{\infty } \\& \quad \leq C_{\mathrm{loc}} \cdot \Delta ^{2} \cdot \Biggl\{ \sum _{j = 0}^{p - 1} \biggl( \frac{1}{1 - 2 \cdot ( \alpha _{\mathrm{max}} + \beta _{\mathrm{max}} ) \cdot \Delta } \biggr)^{j} \Biggr\} \\& \quad = C_{\mathrm{loc}} \cdot \Delta ^{2} \cdot \frac{ ( \frac{1}{1 - 2 \cdot ( \alpha _{\mathrm{max}} + \beta _{\mathrm{max}} ) \cdot \Delta } )^{p} - 1}{ ( \frac{1}{1 - 2 \cdot ( \alpha _{\mathrm{max}} + \beta _{\mathrm{max}} ) \cdot \Delta } ) - 1}. \end{aligned}$$

If we assume $\Delta < \frac{1}{4 \cdot ( \alpha _{\mathrm{max}} + \beta _{\mathrm{max}} )}$, we conclude

$$ \frac{\Delta }{ ( \frac{1}{1 - 2 \cdot ( \alpha _{\mathrm{max}} + \beta _{\mathrm{max}} ) \cdot \Delta } ) - 1} \leq 1, $$

and hence it follows

34 $$ \bigl\lVert \mathbf{z}_{p + 1} - \mathbf{z} ( t_{p + 1} ) \bigr\rVert _{\infty } \leq C_{\mathrm{loc}} \cdot \Delta \cdot \biggl\{ \biggl( \frac{1}{1 - 2 \cdot ( \alpha _{\mathrm{max}} + \beta _{\mathrm{max}} ) \cdot \Delta } \biggr)^{p} - 1 \biggr\} , $$

which finishes our proof of (23). □

### Calculation of the time-discrete basic reproduction number

In our nonautonomous time-discrete SIR model, the time-dependent basic reproduction number can be defined by

35 $$ \mathcal{R}_{0} ( t_{k} ) := \frac{\alpha ( t_{k} )}{\beta ( t_{k} )} $$

for arbitrary $k \in \{ 1, \ldots , M \} $, which is similar to the case of constant transmission and recovery rates [17, 45].

#### Lemma 3

*Equation* (35) *is well defined*.

#### Proof

This proof is identical to Lemma 2. □

### Numerical algorithm

We are now able to give a brief description of our numerical algorithm to solve the time-discrete implicit solution scheme (17). Here, we summarize our inputs, our computational steps, and our algorithmic outputs. We sketch the resulting algorithm in Table 1.

**Table 1:** Numerical algorithm for the time-discrete implicit SIR solution scheme (17)

*Inputs*:	– Population size *N*
	– Initial values $S_{1} > 0$, $I_{1} > 0$, and $R_{1} \geq 0$
	– Time-varying transmission rate sequence $\{ \alpha _{j} \} _{j = 2}^{M}$
	– Time-varying recovery rate sequence $\{ \beta _{j} \} _{j = 2}^{M} $
	– Strictly increasing sequence $\{ t_{j} \} _{j = 1}^{M}$ of time points with $t_{1} = 0$ and $t_{M} = T$
*Step 1*:	– Compute all $\Delta _{j + 1} = t_{j + 1} - t_{j}$ for all *j*∈{1,…,*M* − 1}
*Step 2*:	– Compute $I_{j + 1}$ by (22), (21) and (20) for all *j*∈{1,…,*M* − 1}
	– Compute $S_{j + 1}$ and $R_{j + 1}$ by (17) for all *j*∈{1,…,*M* − 1}
*Outputs*:	– Sequences $\{ S_{j} \} _{j = 1}^{M}$, $\{ I_{j} \} _{j = 1}^{M}$ and $\{ R_{j} \} _{j = 1}^{M}$

## Numerical examples with discussion

We apply our time-discrete implicit SIR solution scheme (22) from Table 1 to available data regarding the spread of COVID-19 in Germany and Iran from John Hopkins University [1, 2]. These countries are chosen because they update confirmed, dead, and estimated recovered cases on a regular basis. In Table 2, we summarize projected population sizes for 2019 from the United Nations [47].

**Table 2:** Projected population sizes for Germany and Iran for 2019

*Country*	Germany	Iran
*Population size*	83,784,000	83,993,000

At first, we consider the example of Germany in detail. We thoroughly describe our approach to check our model’s validity. We only give some simple parameter estimation techniques and vary user-chosen time-dependent parameter functions. Since inverse problems are an active field of research, we refer the readers to works by Bock and Schittkowski for more sophisticated parameter estimation techniques in dynamical systems [48, 49]. Our work also focuses on usefulness in possibly describing real-world data. Afterwards, we state some computational results for data from Iran.

### Description of our approach by the example of Germany

#### Data preprocessing

**Figure 2:**
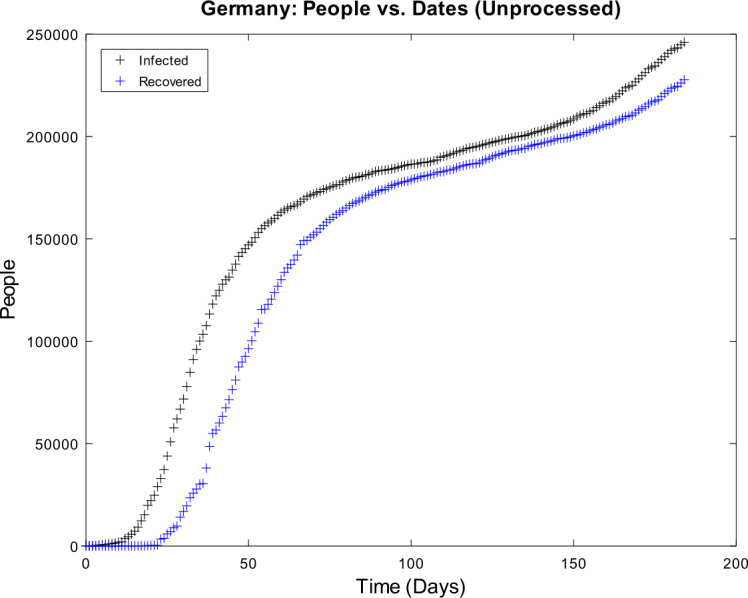
Unprocessed data for Germany with $t_{1} = 0$ (1 March 2020) and $t_{M} = 184$ (1 September 2020)

**Figure 3:**
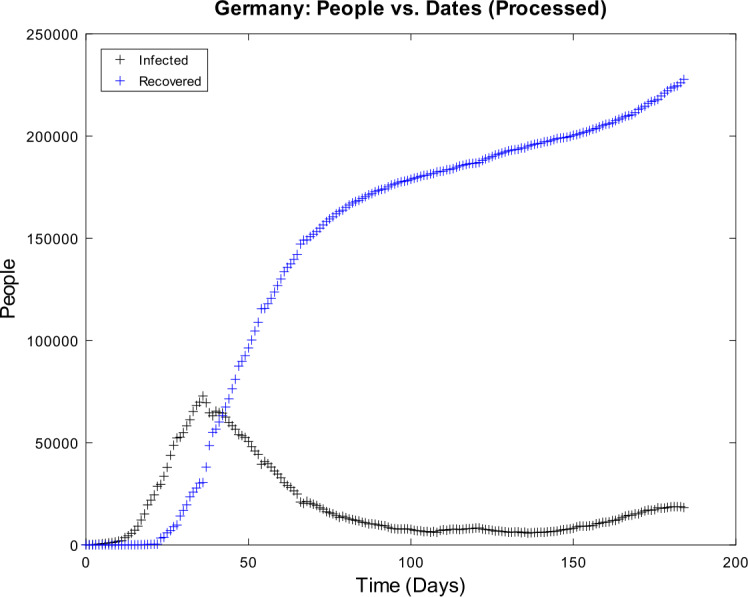
Processed data for Germany with $t_{1} = 0$ (1 March 2020) and $t_{M} = 184$ (1 September 2020)

To apply our model, we have to process the given data of cumulative confirmed infected people $\{ \widetilde{I_{j}} \} _{j = 1}^{M}$, cumulative confirmed dead people $\{ \widetilde{D_{j}} \} _{j = 1}^{M}$, and cumulative confirmed recovered people $\{ \widetilde{R_{j}} \} _{j = 1}^{M}$. For our model, we need to compute the processed real-world data

36 $$ \textstyle\begin{cases} \widetilde{\widetilde{R_{j}}} = \widetilde{R_{j}} + \widetilde{D_{j}}, \\ \widetilde{\widetilde{I_{j}}} = \widetilde{I_{j}} - \widetilde{\widetilde{R_{j}}}, \\ \widetilde{\widetilde{S_{j}}} = N - \widetilde{\widetilde{I_{j}}} - \widetilde{\widetilde{R_{j}}} \end{cases} $$

for all $j \in \{ 1, \ldots , M \} $. The unprocessed and processed data for Germany are depicted in Figs. 2 and 3. On the one hand, it can be clearly seen in Fig. 2 that both sequences of cumulative infected and cumulative recovered people are monotonically increasing for our unprocessed data. On the other hand, we notice in Fig. 3 that the behavior changes for our processed data.

#### Calculation of time-varying transmission and recovery rates from real-world data

Here, we present an algorithm to calculate our time-varying transmission and recovery rates based on our numerical algorithm (15) for all $j \in \{ 1, \ldots , M - 1 \} $. We rely on the first equation and the last equation for susceptible and recovered people. Short calculations with the assumptions $\widetilde{\widetilde{I_{j + 1}}} \neq 0$ and $\widetilde{\widetilde{S_{j + 1}}} \neq 0$ yield

$$\begin{aligned} \widetilde{\widetilde{\alpha _{j + 1}}} = & - \frac{N}{\widetilde{\widetilde{I_{j + 1}}} \cdot \widetilde{\widetilde{S_{j + 1}}}} \cdot \frac{\widetilde{\widetilde{S_{j + 1}}} - \widetilde{\widetilde{S_{j}}}}{\Delta _{j + 1}} \\ = & \frac{N}{\widetilde{\widetilde{I_{j + 1}}} \cdot \widetilde{\widetilde{S_{j + 1}}}} \cdot \frac{\widetilde{\widetilde{S_{j}}} - \widetilde{\widetilde{S_{j + 1}}}}{\Delta _{j + 1}} \\ \geq & 0 \end{aligned}$$

and

$$ \widetilde{\widetilde{\beta _{j + 1}}} = \frac{1}{\widetilde{\widetilde{I_{j + 1}}}} \cdot \frac{\widetilde{\widetilde{R_{j + 1}}} - \widetilde{\widetilde{R_{j}}}}{\Delta _{j + 1}} \geq 0. $$

Summarizing our results, we obtain

37 $$ \widetilde{\widetilde{\alpha _{j + 1}}} = \frac{N}{\widetilde{\widetilde{I_{j + 1}}} \cdot \widetilde{\widetilde{S_{j + 1}}}} \cdot \frac{\widetilde{\widetilde{S_{j}}} - \widetilde{\widetilde{S_{j + 1}}}}{\Delta _{j + 1}} $$

and

38 $$ \widetilde{\widetilde{\beta _{j + 1}}} = \frac{1}{\widetilde{\widetilde{I_{j + 1}}}} \cdot \frac{\widetilde{\widetilde{R_{j + 1}}} - \widetilde{\widetilde{R_{j}}}}{\Delta _{j + 1}}, $$

and a short algorithmic summary can be found in Table 3.

**Table 3:** Numerical algorithm for the time-discrete implicit SIR solution scheme (17)

*Inputs*:	– Population size *N*
	– Real-world data $\{ \widetilde{\widetilde{S_{j}}} \} _{j = 1}^{M}$, $\{ \widetilde{\widetilde{I_{j}}} \} _{j = 1}^{M}$, and $\{ \widetilde{\widetilde{R_{j}}} \} _{j = 1}^{M}$ according to (36)
	– Strictly increasing sequence $\{ t_{j} \} _{j = 1}^{M}$ of time points with $t_{1} = 0$ and $t_{M} = T$
*Step 1*:	– Compute all $\Delta _{j + 1} = t_{j + 1} - t_{j}$ for all *j*∈{1,…,*M* − 1}
*Step 2*:	– For all *j*∈{2,…,*M*}, compute $\widetilde{\widetilde{\alpha _{j}}}$ and $\widetilde{\widetilde{\beta _{j}}}$ according to (37) and (38) with real-world data
*Outputs*:	– Sequences $\{ \widetilde{\widetilde{\alpha _{j}}} \} _{j = 2}^{M}$ and $\{ \widetilde{\widetilde{\beta _{j}}} \} _{j = 2}^{M}$

**Figure 4:**
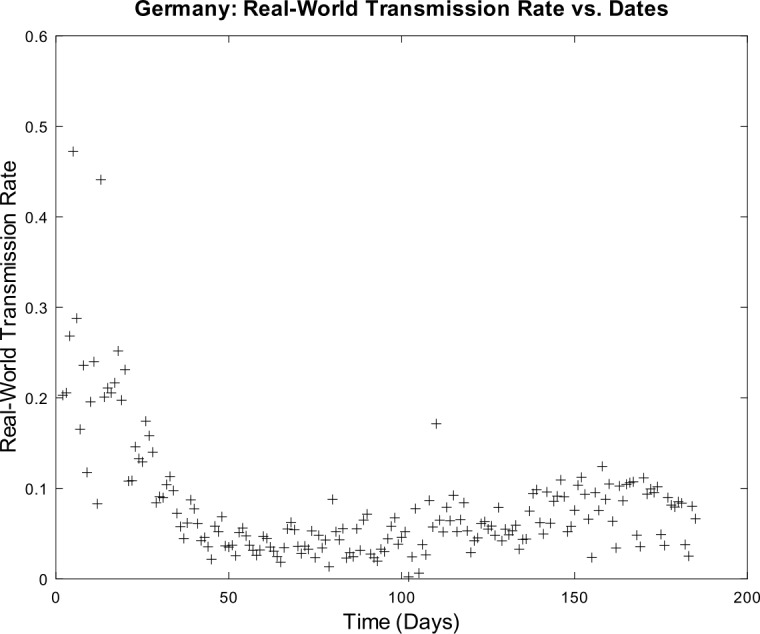
Time-varying transmission rate from real-world data for Germany with $t_{1} = 0$ (1 March 2020) and $t_{M} = 184$ (1 September 2020)

The time-varying transmission rate from real-world data for Germany is presented in Fig. 4. Clearly, the transmission rate decreases due to countermeasures such as local lock-downs and voluntary social distancing by the population. However, a weak increasing trend can be seen at the end of the time-series in August. Possible explanations might be opening of schools, universities or people who do not wear masks for protection.

**Figure 5:**
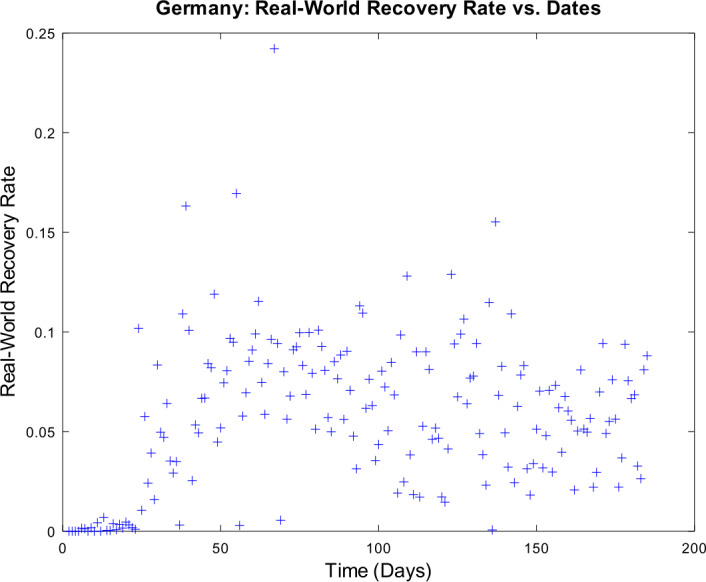
Time-varying recovery rate from real-world data for Germany with $t_{1} = 0$ (1 March 2020) and $t_{M} = 184$ (1 September 2020)

The time-varying recovery rate from real-world data for Germany is depicted in Fig. 5. At the early stage of an epidemic, there are possible just few recoveries, thus this rate is relatively small. After some time, this situation changes as more people defeat the disease and recover. The rate seems to be constant with heavy variations due to the test capacity. Additionally, there are unknown cases because these people might have a mild disease course.

#### Calculation of time-dependent basic reproduction number from real-world data

**Figure 6:**
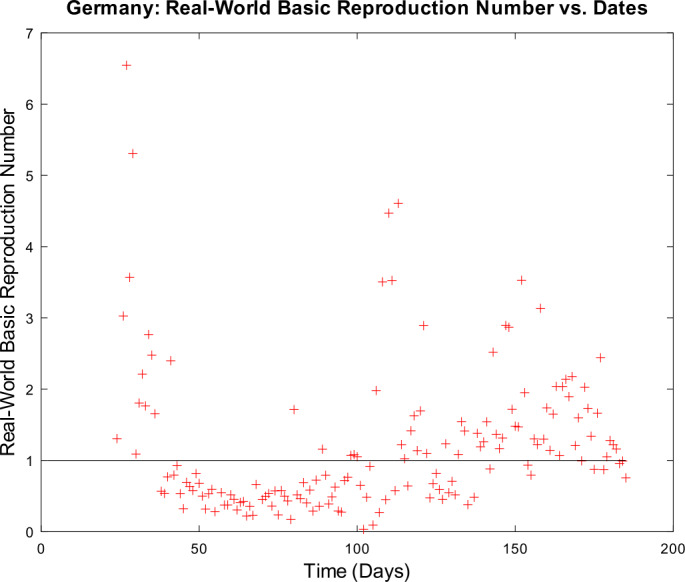
Time-varying basic reproduction number from real-world data for Germany with $t_{1} = 0$ (1 March 2020) and $t_{M} = 184$ (1 September 2020)

Now, the time-dependent basic reproduction number $\mathcal{R}_{0} ( t_{j} )$ is readily computed by (35). Our computational results from this approach are portrayed in Fig. 6. Since there are only few recovered people at the beginning of disease, our computations provide high numerical basic reproduction number. We observe that the computational basic reproduction number is monotonically decreasing in spring due to political countermeasures and social distancing. However, the graph shows that the computation basic reproduction number rises in summer because contacts between people rose. Variations are seen for similar reasons as mentioned for our time-dependent transmission and recovery rates.

#### Parameter estimation—a simple least-squares approach

We only briefly sketch the parameter identification problem because it is an inverse problem [50, 51]. A deep discussion is beyond the scope of this paper and would be a topic of own interest. By looking at Figs. 4 and 5, we assume

39 $$ \alpha ( t ) := \alpha _{1} \cdot \exp ( - \alpha _{t_{2}} \cdot t ) $$

and

40 $$ \beta ( t ) := \beta $$

with real constants $\alpha _{1}$, $\alpha _{2}$, and *β* which we determine from real-world transmission and recovery rate sequences $\{ \widetilde{\widetilde{\alpha _{j}}} \} _{j = 2}^{M}$ and $\{ \widetilde{\widetilde{\beta _{j}}} \} _{j = 2}^{M}$. Since $\widetilde{\widetilde{\alpha _{j}}} > 0$ and $\widetilde{\widetilde{\beta _{j}}} > 0$ for all $j \in \{ 2, \ldots , M \} $, we can assume $\alpha _{1} > 0$ and $\beta > 0$. Since $\alpha ( t )$ is nonlinear, we use the transformation

41 $$ \ln \bigl( \alpha ( t ) \bigr) = \ln ( \alpha _{1} ) - \alpha _{2} \cdot t = \gamma _{1} + \gamma _{2} \cdot t $$

with $\gamma _{1} := \ln ( \alpha _{1} )$ and $\gamma _{2} := - \alpha _{2}$ as in the case of maximum log-likelihood estimation. Now, our cost function $\mathcal{J} \colon \mathbb{R}^{3} \longrightarrow [ 0, \infty )$ reads as follows:

42 $$ \mathcal{J} ( \gamma _{1}, \gamma _{2}, \beta ) := \sum_{j = 2}^{M} \bigl( \gamma _{1} + \gamma _{2} \cdot t_{j} - \ln ( \widetilde{\widetilde{\alpha _{j}}} ) \bigr)^{2} + \sum_{j = 2}^{M} ( \beta - \widetilde{\widetilde{\beta _{j}}} )^{2}. $$

We obtain the following theorem.

##### Theorem 12

*Assume that*

$$ \sum_{j = 2}^{M} t_{j}^{2} - \frac{1}{M - 1} \cdot \Biggl( \sum_{j = 1}^{M} t_{j} \Biggr)^{2} > 0 $$

*holds for the strictly increasing time sequence* $\{ t_{j} \} _{j = 1}^{M}$. *The cost function* (42) *possesses a unique local minimizer*. *In fact*, *this unique local minimizer is even a unique global minimizer*.

##### Proof

1) We first show that *J* possesses a unique local minimizer.

1.1) To achieve our goal, we calculate the first derivatives. We obtain

$$\begin{aligned}& \frac{\partial J}{\partial \gamma _{1}} ( \gamma _{1}, \gamma _{2}, \beta ) = 2 \cdot \sum_{j = 2}^{M} \bigl\{ \gamma _{1} + \gamma _{2} \cdot t_{j} - \ln ( \widetilde{\widetilde{\alpha _{j}}} ) \bigr\} , \\& \frac{\partial J}{\partial \gamma _{2}} ( \gamma _{1}, \gamma _{2}, \beta ) = 2 \cdot \sum_{j = 2}^{M} t_{j} \cdot \bigl\{ \gamma _{1} + \gamma _{2} \cdot t_{j} - \ln ( \widetilde{\widetilde{\alpha _{j}}} ) \bigr\} , \\& \frac{\partial J}{\partial \beta } ( \gamma _{1}, \gamma _{2}, \beta ) = 2 \cdot \sum_{j = 2}^{M} \{ \beta - \widetilde{\widetilde{\beta _{j}}} \} . \end{aligned}$$

1.2) To get a local minimizer $( \widehat{\gamma _{1}}, \widehat{\gamma _{2}}, \widehat{\beta } )$, it is necessary that all partial derivatives vanish at candidates for local extrema.

1.2a) Setting $\frac{\partial J}{\partial \beta } ( \widehat{\gamma _{1}}, \widehat{\gamma _{2}}, \widehat{\beta } ) = 0$, we conclude

43 $$ \widehat{\beta } = \frac{1}{M - 1} \cdot \sum _{j = 2}^{M} \widetilde{\widetilde{\beta _{j}}}. $$

1.2b) Setting $\frac{\partial J}{\partial \gamma _{1}} ( \widehat{\gamma _{1}}, \widehat{\gamma _{2}}, \widehat{\beta } ) = 0$, we infer that

44 $$ \widehat{\gamma _{1}} = \frac{1}{M - 1} \cdot \Biggl\{ \sum_{j = 2}^{M} \bigl( \ln ( \widetilde{\widetilde{\alpha _{j}}} ) - t_{j} \cdot \widehat{\gamma _{2}} \bigr) \Biggr\} $$

holds.

1.2c) If we set $\frac{\partial J}{\partial \gamma _{2}} ( \widehat{\gamma _{1}}, \widehat{\gamma _{2}}, \widehat{\beta } ) = 0$, we obtain

$$\begin{aligned}& \widehat{\gamma _{2}} \cdot \Biggl( \sum _{j = 2}^{M} t_{j}^{2} \Biggr) \\& \quad = \sum_{j = 2}^{M} \bigl\{ t_{j} \cdot \ln ( \widetilde{\widetilde{\alpha _{j}}} ) \bigr\} - \gamma _{1} \cdot \sum_{j = 2}^{M} t_{j} \\& \quad = \sum_{j = 2}^{M} \bigl\{ t_{j} \cdot \ln ( \widetilde{\widetilde{\alpha _{j}}} ) \bigr\} - \Biggl\{ \frac{1}{M - 1} \cdot \Biggl\{ \sum _{j = 2}^{M} \ln ( \widetilde{\widetilde{\alpha _{j}}} ) - \widehat{\gamma _{2}} \cdot \sum _{j = 2}^{M} t_{j} \Biggr\} \Biggr\} \cdot \Biggl\{ \sum_{j = 2}^{M} t_{j} \Biggr\} \end{aligned}$$

and this yields

$$\begin{aligned}& \widehat{\gamma _{2}} \cdot \Biggl\{ \Biggl( \sum _{j = 2}^{M} t_{j}^{2} \Biggr) - \frac{1}{M - 1} \cdot \Biggl( \sum_{j = 2}^{M} t_{j} \Biggr)^{2} \Biggr\} \\& \quad = \Biggl( \sum_{j = 2}^{M} \bigl\{ t_{j} \cdot \ln ( \widetilde{\widetilde{\alpha _{j}}} ) \bigr\} \Biggr) - \frac{1}{M - 1} \cdot \Biggl\{ \sum _{j = 2}^{M} \ln ( \widetilde{\widetilde{\alpha _{j}}} ) \Biggr\} \cdot \Biggl\{ \sum_{j = 2}^{M} t_{j} \Biggr\} . \end{aligned}$$

Finally, we conclude that

45 $$ \widehat{\gamma _{2}} = \frac{ ( \sum_{j = 2}^{M} \{ t_{j} \cdot \ln ( \widetilde{\widetilde{\alpha _{j}}} ) \} ) - \frac{1}{M - 1} \cdot \{ \sum_{j = 2}^{M} \ln ( \widetilde{\widetilde{\alpha _{j}}} ) \} \cdot \{ \sum_{j = 2}^{M} t_{j} \} }{ \{ ( \sum_{j = 2}^{M} t_{j}^{2} ) - \frac{1}{M - 1} \cdot ( \sum_{j = 2}^{M} t_{j} )^{2} \} } $$

holds.

1.3) Since the Hessian is given by

$$ H ( \gamma _{1}, \gamma _{2}, \beta ) = \begin{pmatrix} 2 \cdot ( M - 1 ) & 2 \cdot \sum_{j = 2}^{M} t_{j} & 0 \\ 2 \cdot \sum_{j = 2}^{M} t_{j} & 2 \cdot \sum_{j = 2}^{M} t_{j}^{2} & 0 \\ 0 & 0 & 2 \cdot ( M - 1 ) \end{pmatrix} , $$

we investigate all determinants of all upper-left sub-matrices. The first determinant $2 \cdot ( M - 1 ) > 0$ because $M \geq 2$ holds. The second determinant reads as follows:

$$ 4 \cdot ( M - 1 ) \cdot \sum_{j = 2}^{M} - 4 \cdot \Biggl( \sum_{j = 2}^{M} t_{j} \Biggr)^{2}. $$

By the Hölder inequality, we obtain

$$ 4 \cdot ( M - 1 ) \cdot \sum_{j = 2}^{M} t_{j}^{2} - 4 \cdot \Biggl( \sum _{j = 2}^{M} t_{j} \Biggr)^{2} \geq 0 $$

with equality only in the case that all $t_{j}$ are equal. Since we have a strictly increasing time sequence, the second determinant of the upper-left sub-matrices is also positive. Finally, the determinant of the full matrix is positive as well. Hence, our cost function *J* is strictly convex. Conclusively, it possesses a unique local minimizer by [52, Theorem 2.4].

2) By strict convexity, we infer that the unique local minimizer is also the unique global minimizer of our cost function *J* by [52, Theorem 2.5]. □

We summarize our algorithmic approach for parameter estimation of our time-varying transmission and recovery rates in Table 4.

**Table 4:** Numerical algorithm for our parameter estimation approach of our cost function (42)

*Inputs*:	– Strictly increasing sequence $\{ t_{j} \} _{j = 1}^{M}$ of time points with $t_{1} = 0$ and $t_{M} = T$
	– Sequences $\{ \widetilde{\widetilde{\alpha _{j}}} \} _{j = 2}^{M}$ and $\{ \widetilde{\widetilde{\beta _{j}}} \} _{j = 2}^{M}$
*Step 1*:	– Compute *β̂* by (43)
*Step 2*:	– Compute $\widehat{\gamma _{2}}$ by (45)
*Step 3*:	– Compute $\widehat{\gamma _{1}}$ by (44)
*Step 4*:	– Compute $\widehat{\alpha _{1}}$ and $\widehat{\alpha _{2}}$ according to transformation (41)
*Outputs*:	– Parameters $\widehat{\alpha _{1}}$, $\widehat{\alpha _{2}}$, and *β̂* for our parametric rates (39) and (40)

#### Results for our parameter estimation approach using German data for short-term predictions

**Figure 7:**
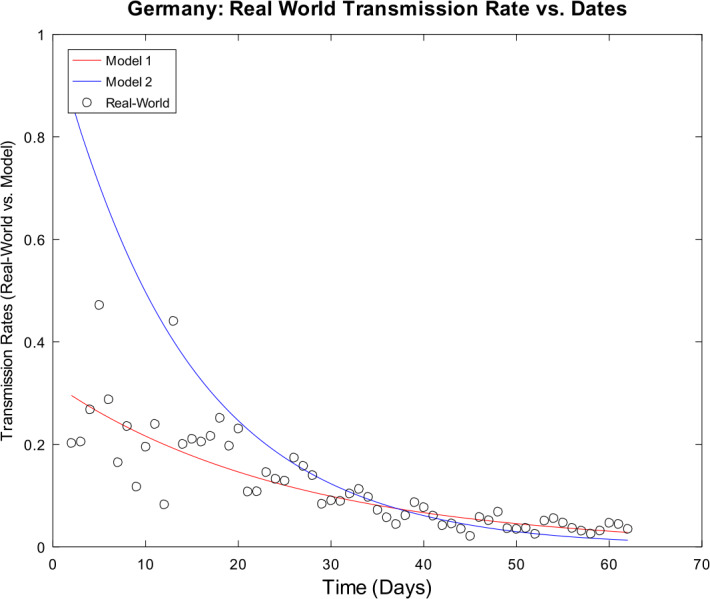
Time-varying transmission rates from real-world data and from parameter estimation for short-term prediction of German data with $t_{1} = 0$ (1 March 2020) and $t_{M} = 62$ (2 May 2020). The estimated parameters for model 1 are $\alpha _{1} \approx 0.3194$ and $\alpha _{2} \approx 0.003911$. For model 2, we fix $\alpha _{1} = 1.0$ and choose $\alpha _{2} \approx 0.070$

**Figure 8:**
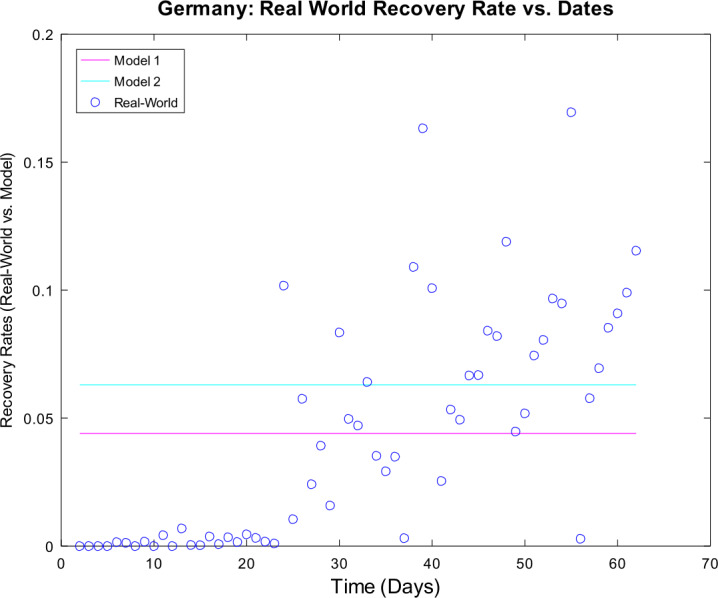
Recovery rates from real-world data and from parameter estimation for short-term prediction of German data with $t_{1} = 0$ (1 March 2020) and $t_{M} = 62$ (2 May 2020). The first estimated recovery rate reads $\beta \approx 0.04403$ for the mean value on the full interval. The second estimated recovery rate reads $\beta \approx 0.063$ as the mean value on the time interval $[ 25, 62 ]$ because the fluctuations in *β* arise while there is no recovery rate estimation possible for the first days

**Figure 9:**
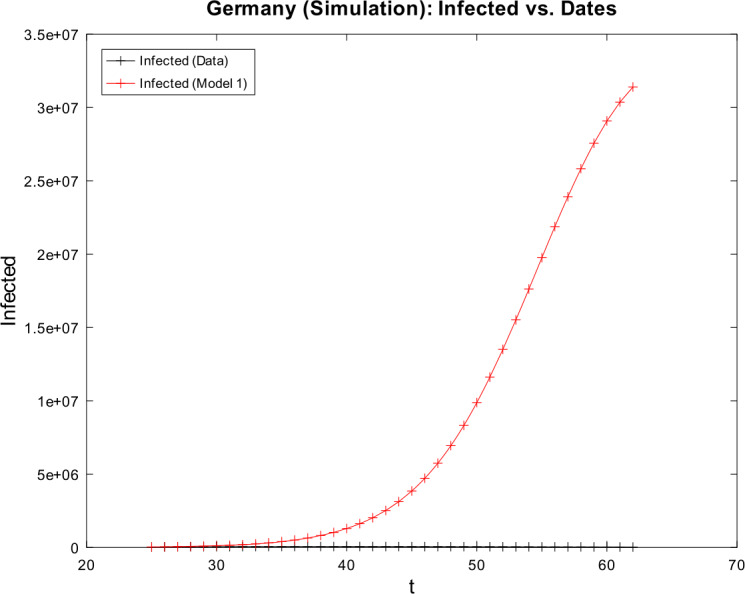
Computational results for model 1 of infected people in Germany for $\alpha _{1} \approx 0.3194$, $\alpha _{2} \approx 0.003911$, $\beta \approx 0.063$ for short-time simulation on $t_{1} = 25$ (26 March 2020) and $t_{M} = 62$ (2 May 2020) with real-world data as initial conditions

**Figure 10:**
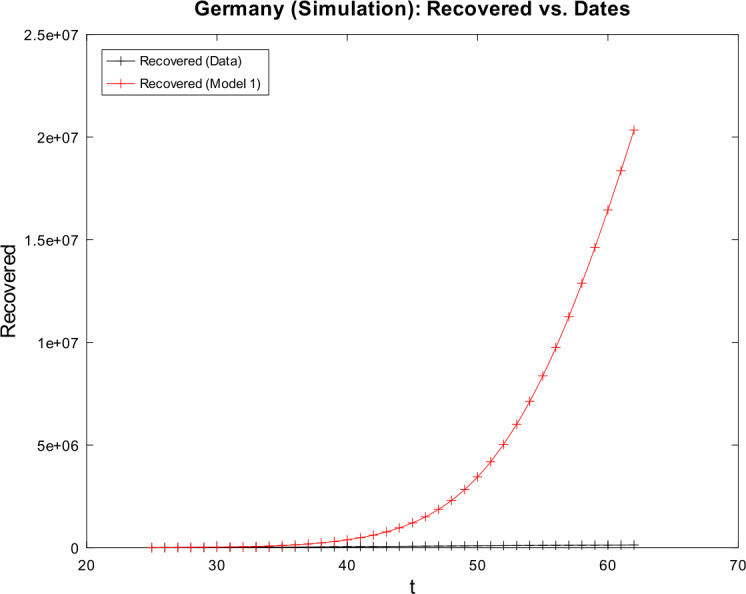
Computational results for model 1 of recovered people in Germany for $\alpha _{1} \approx 0.3194$, $\alpha _{2} \approx 0.003911$, $\beta \approx 0.063$ for short-time simulation on $t_{1} = 25$ (26 March 2020) and $t_{M} = 62$ (2 May 2020) with real-world data as initial conditions

**Figure 11:**
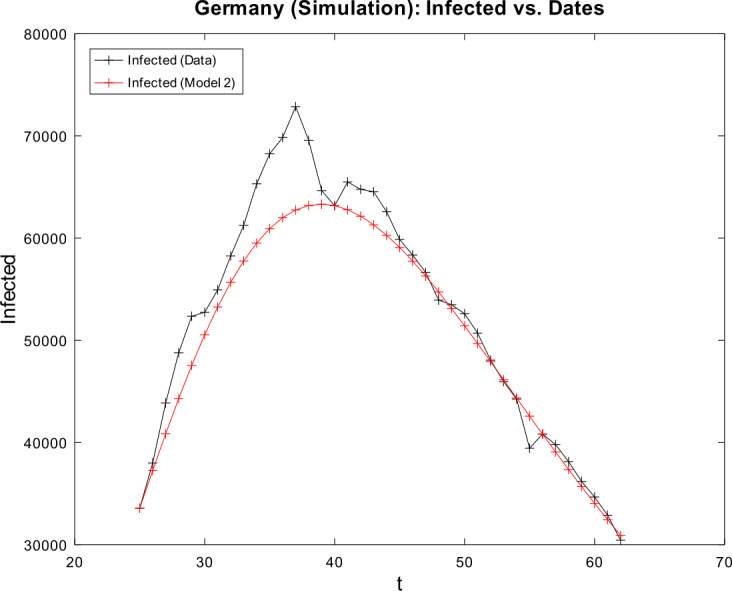
Computational results for model 2 of infected people in Germany for $\alpha _{1} \approx 1.0$, $\alpha _{2} \approx 0.070$, $\beta \approx 0.063$ for short-time simulation on $t_{1} = 25$ (26 March 2020) and $t_{M} = 62$ (2 May 2020) with real-world data as initial conditions

**Figure 12:**
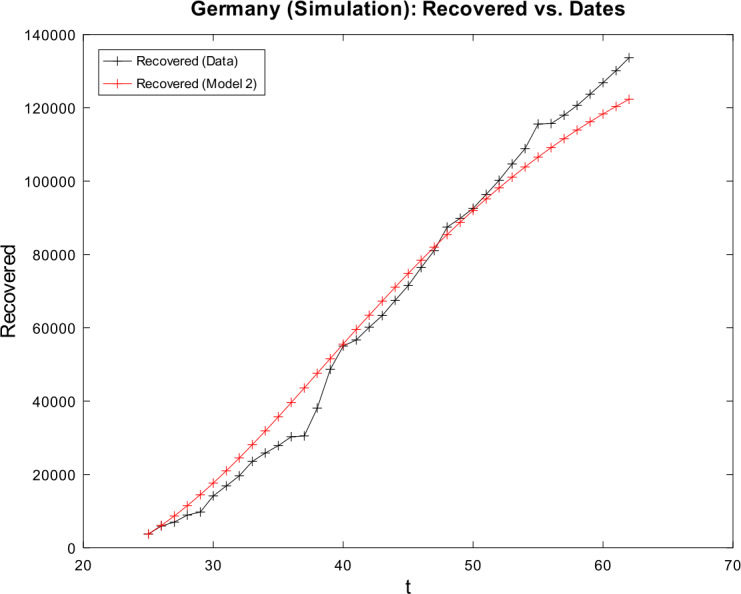
Computational results for model 2 of recovered people in Germany for $\alpha _{1} \approx 1.0$, $\alpha _{2} \approx 0.070$, $\beta \approx 0.063$ for short-time simulation on $t_{1} = 25$ (26 March 2020) and $t_{M} = 62$ (2 May 2020) with real-world data as initial conditions

In Fig. 7, we see that the assumption of exponentially decaying time-dependent transmission rates is acceptable at the beginning of spreading disease with respect to German data. Due to short-term prediction, we notice that the constant recovery rate is underestimated in Fig. 8. Conclusively, both assumptions seem to be acceptable at the first weeks of a spreading disease. Computational results for two models on the time interval $[ 25, 62 ]$ are depicted in Figs. 9–12. Figures 9–12 indicate that sensitivity of parameters is really an issue in epidemiological models. This is in accordance with Theorem 7. These results also imply that an exponentially decaying transmission rate is an acceptable choice at the beginning of spreading disease.

#### Results for our parametric approach using German data from May to September

**Figure 13:**
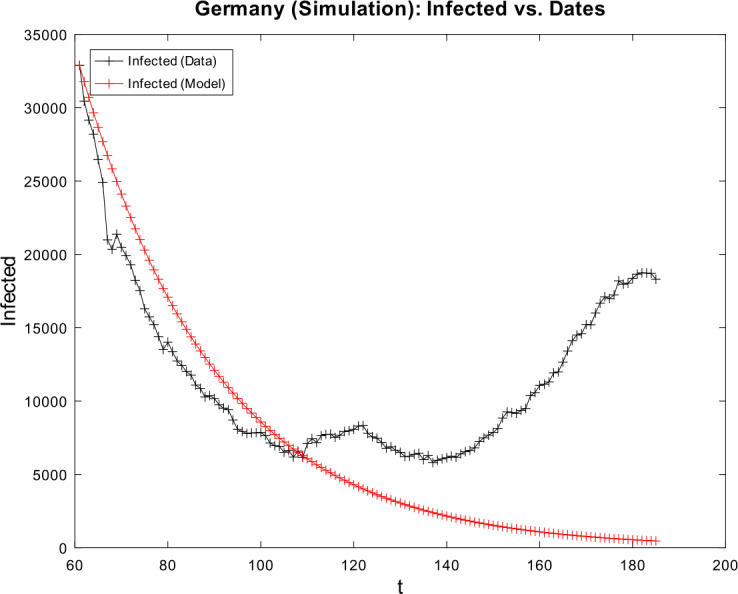
Computational results for our model of infected people in Germany for user-chosen $\alpha _{1} \approx 0.040$, $\alpha _{2} \approx 0.0$, $\beta \approx 0.075$ for short-time simulation on $t_{1} = 61$ (1 May 2020) and $t_{M} = 184$ (1 September 2020) with real-world data as initial conditions

**Figure 14:**
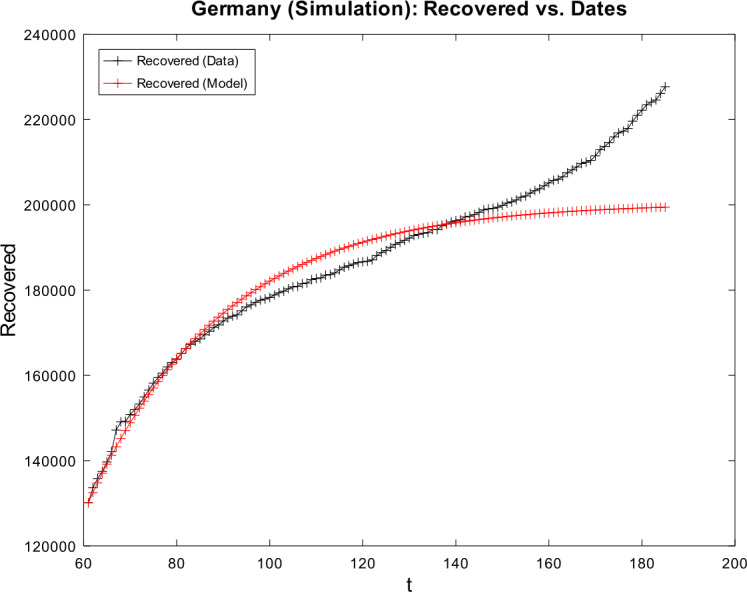
Computational results for our model of recovered people in Germany for user-chosen $\alpha _{1} \approx 0.040$, $\alpha _{2} \approx 0.0$, $\beta \approx 0.075$ for short-time simulation on $t_{1} = 61$ (1 May 2020) and $t_{M} = 184$ (1 September 2020) with real-world data as initial conditions

Figures 4 and 5 indicate that constant transmission and recovery rates are reasonable assumption at later stages. Computational results can be found in Figs. 13 and 14. We also notice that the number of infected people rises in summer, possibly due to more social contacts. This could eventually be regarded as the beginning of a ‘second wave’.

For future investigations, it might be interesting to use more complex transmission rates or piecewise defined functions with switching points, see e.g. [49].

### Computational short-time results for data from Iran

**Figure 15:**
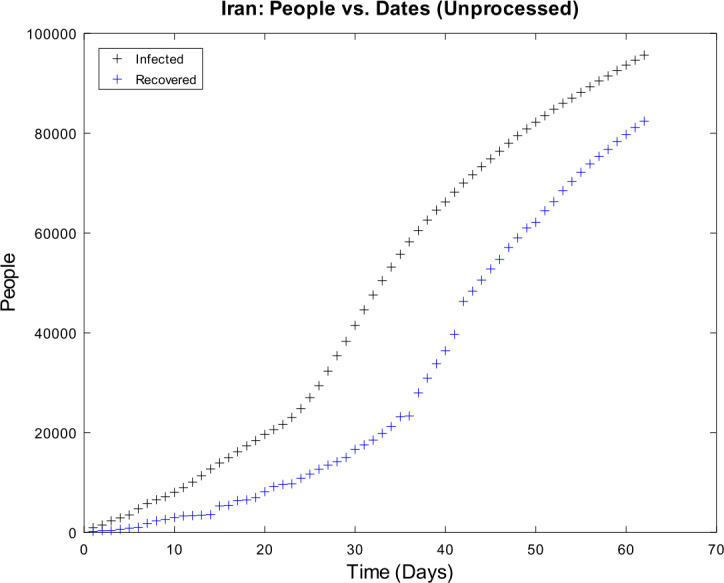
Unprocessed data for Iran with $t_{1} = 1$ (1 March 2020) and $t_{M} = 62$ (1 May 2020)

**Figure 16:**
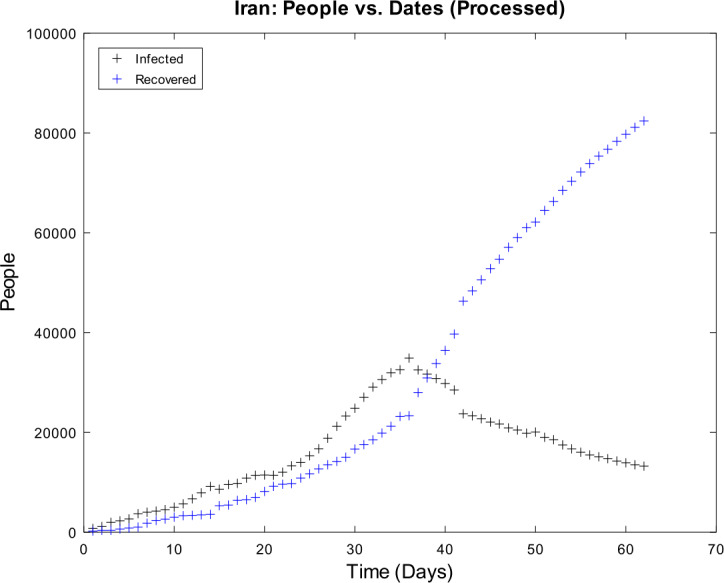
Processed data for Iran with $t_{1} = 1$ (1 March 2020) and $t_{M} = 62$ (1 May 2020)

**Figure 17:**
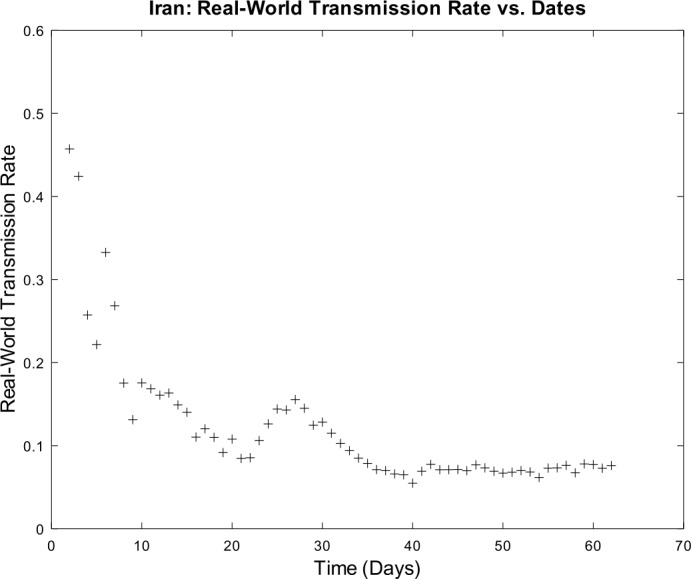
Time-varying transmission rate from real-world data for Iran with $t_{1} = 1$ (1 March 2020) and $t_{M} = 62$ (1 May 2020)

**Figure 18:**
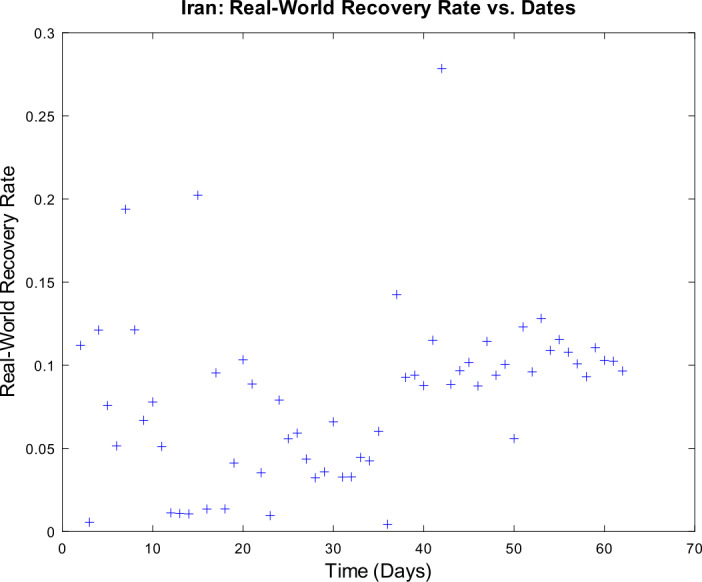
Time-varying recovery rate from real-world data for Iran with $t_{1} = 1$ (1 March 2020) and $t_{M} = 62$ (1 May 2020)

**Figure 19:**
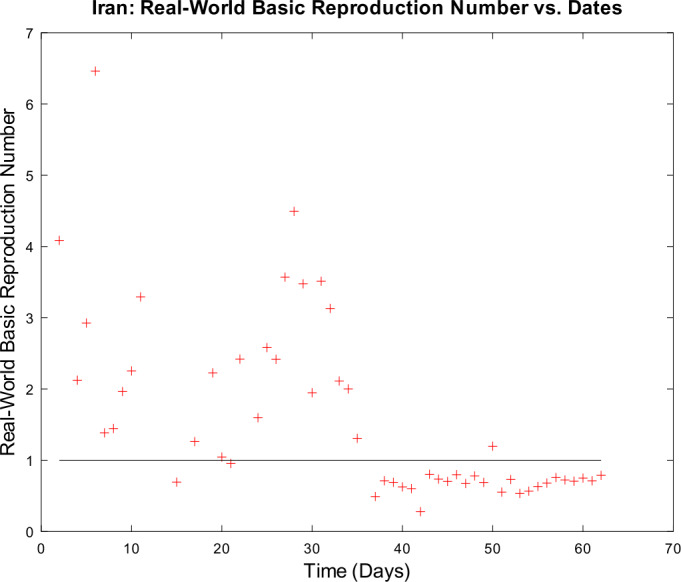
Time-varying basic reproduction number from real-world data for Iran with $t_{1} = 1$ (1 March 2020) and $t_{M} = 62$ (1 May 2020)

**Figure 20:**
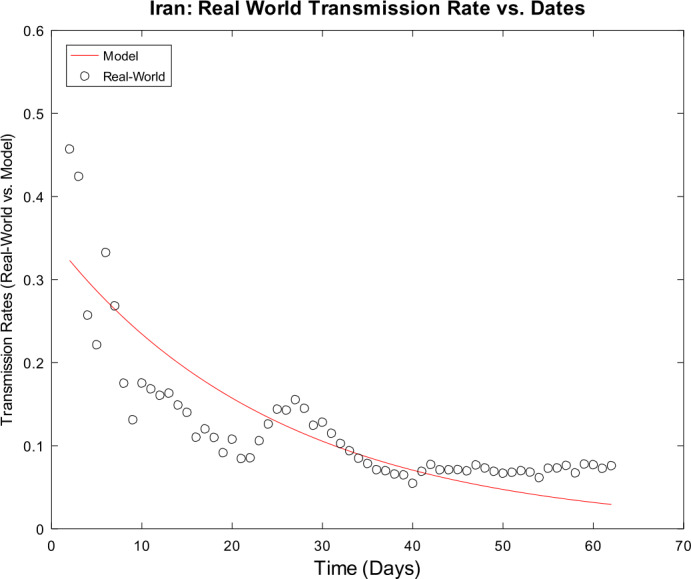
Time-varying transmission rates from real-world data for data from Iran with $t_{1} = 1$ (1 March 2020) and $t_{M} = 62$ (1 May 2020). The user-chosen parameters for our model are $\alpha _{1} \approx 0.350$ and $\alpha _{2} \approx 0.040$

**Figure 21:**
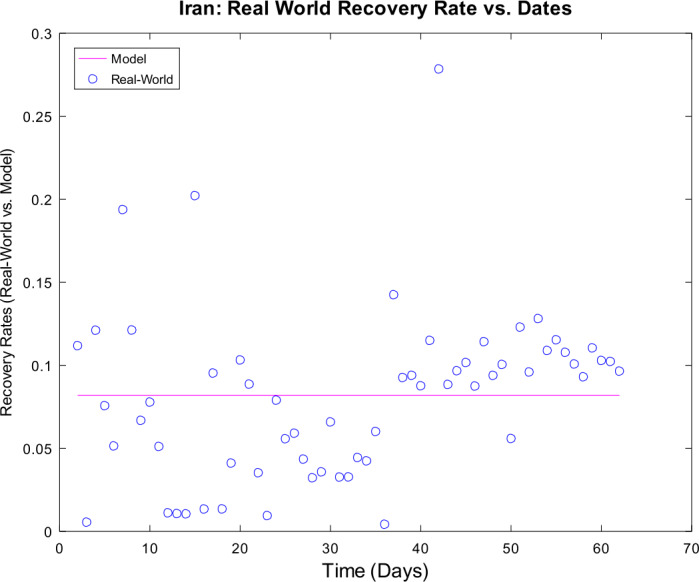
Recovery rates from real-world data for data from Iran with $t_{1} = 1$ (1 March 2020) and $t_{M} = 62$ (2 May 2020). The user-chosen recovery rate reads $\beta \approx 0.082$

**Figure 22:**
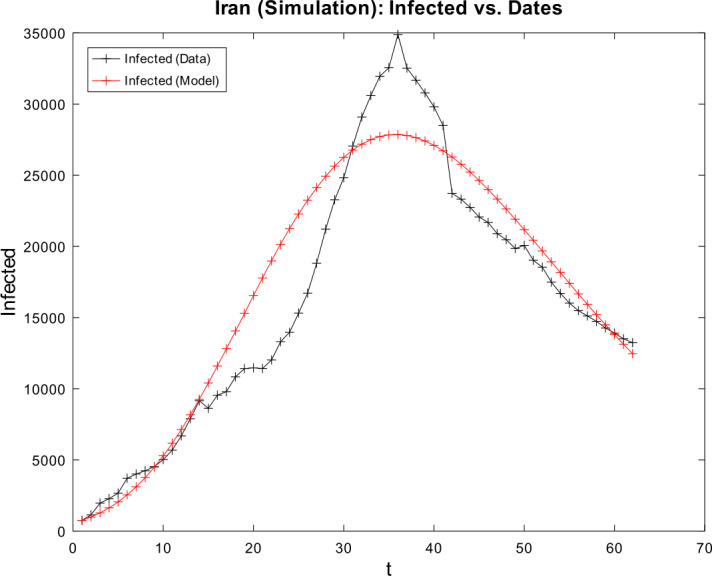
Computational results for our model with transmission and recovery rates from Figs. 20 and 21 of infected people in Iran for short-time simulation on $t_{1} = 1$ (1 March 2020) and $t_{M} = 62$ (1 May 2020) with real-world data as initial conditions

**Figure 23:**
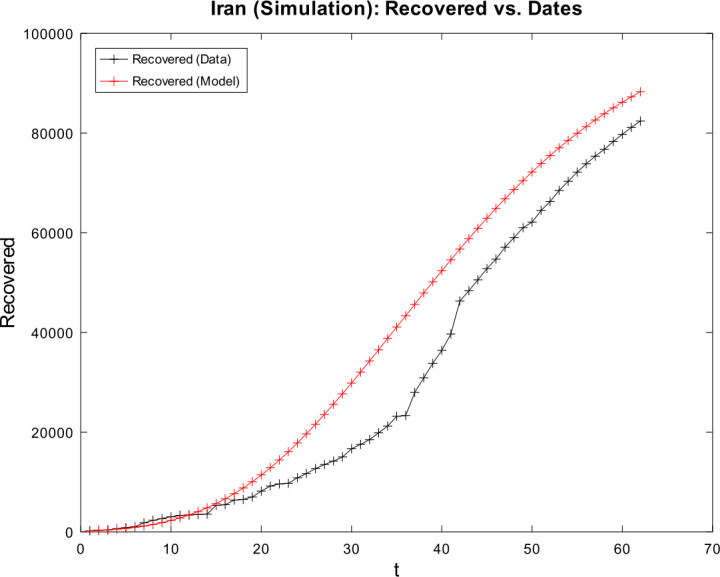
Computational results for our model with transmission and recovery rates from Figs. 20 and 21 of recovered people in Iran for short-time simulation on $t_{1} = 1$ (1 March 2020) and $t_{M} = 62$ (1 May 2020) with real-world data as initial conditions

Real-world data from Iran and short-term computational results for COVID-19 data from Iran can be found in Figs. 15–23. Figure 17 again supports the assumption of an exponentially decaying transmission rate at the beginning of the spread of life-threatening disease. The computation results, depicted in Figs. 22 and 23, show qualitative agreement with the trends in real-world data. These results indicate that time-dependent transmission rates are a necessary addition to the classical SIR model. Alternatively, models with fractional derivatives could be considered [53, 54].

## Conclusion and outlook

We established certain properties such as well-posedness of the solution of our time-continuous SIR model in Sect. 2. Fortunately, we were able to transfer many properties of the time-continuous model to our time-discrete implicit SIR model in Sect. 3. These include unique solvability and monotonicity properties. In contrast to many other works mentioned in Sect. 1, we avoid an explicit forward model, but we could transform our implicit scheme to an easily solvable scheme. Thus, this makes our proposed scheme an attractive first prediction choice. In addition to that, we showed that our numerical scheme possesses an upper error bound.

Regarding our computational results, we see that our parametrization

$$ \alpha ( t ) = \alpha _{1} \cdot \exp ( - \alpha _{2} \cdot t ) $$

is an appropriate fit for first forecasts considering the first wave of a spreading virus. Since these transmission rates are monotonically decreasing, we, however, remark that we will need to use another parametrization if we want to model diseases with seasonal behavior [55]. Regarding our chosen examples, we get reasonable results. Additionally, we observe that our theoretical findings regarding monotonicity of recovered people from Theorem 10 are fulfilled in both examples. This stresses the attractiveness of our implicit solution scheme.

As depicted in Sect. 4, the inverse problem definitely needs further investigation. This is a topic of its own interest [56–59] since we need tools from different mathematical disciplines. As future research directions, extensions to further epidemiological forward models should be considered as we surely need more tools to predict the impact of upcoming epidemics. One can also consider delayed-differential or stochastic variants of our SIR model or modifications and extensions [23, 27] because, from a biological point of view, we often have to face integration of incubation times in epidemic models.
